# The Regulation of Plant Secondary Metabolism in Response to Abiotic Stress: Interactions Between Heat Shock and Elevated CO_2_

**DOI:** 10.3389/fpls.2019.01463

**Published:** 2019-11-14

**Authors:** Nichola Austen, Heather J. Walker, Janice Ann Lake, Gareth K. Phoenix, Duncan Drummond Cameron

**Affiliations:** Department of Animal and Plant Sciences, University of Sheffield, Sheffield, United Kingdom

**Keywords:** metabolism, flavonoids, climate change, isoprene, mass spectrometry, abiotic stress, willow

## Abstract

Future climate change is set to have an impact on the physiological performance of global vegetation. Increasing temperature and atmospheric CO_2_ concentration will affect plant growth, net primary productivity, photosynthetic capability, and other biochemical functions that are essential for normal metabolic function. Alongside the primary metabolic function effects of plant growth and development, the effect of stress on plant secondary metabolism from both biotic and abiotic sources will be impacted by changes in future climate. Using an untargeted metabolomic fingerprinting approach alongside emissions measurements, we investigate for the first time how elevated atmospheric CO_2_ and temperature both independently and interactively impact on plant secondary metabolism through resource allocation, with a resulting “trade-off” between secondary metabolic processes in *Salix* spp. and in particular, isoprene biosynthesis. Although it has been previously reported that isoprene is suppressed in times of elevated CO_2_, and that isoprene emissions increase as a response to short-term heat shock, no study has investigated the interactive effects at the metabolic level. We have demonstrated that at a metabolic level isoprene is still being produced during periods of both elevated CO_2_ and temperature, and that ultimately temperature has the greater effect. With global temperature and atmospheric CO_2_ concentrations rising as a result of anthropogenic activity, it is imperative to understand the interactions between atmospheric processes and global vegetation, especially given that global isoprene emissions have the potential to contribute to atmospheric warming mitigation.

## Introduction

Resisting environmental stress is one of the major challenges faced by plants. A central mechanism deployed by plants that allows them to mitigate environmental stress is the modulation of their secondary metabolism. For example, this can take the form of anti-feedant compounds to reduce herbivory (Hopkins et al., 2017), and the production of compatible solutes that allow plants to maintain homeostatic water relations under drought conditions (Holström et al., 2000). Such environmental challenges are now being exacerbated by climate change, and therefore, there is a need to understand the biological controls on secondary metabolism in relation to changing climate. This is critical in order to make predictions about potential responses of global vegetation to future climatic scenarios.

Extreme temperature, as well as environmental factors such as increased atmospheric CO_2_ concentrations are some of the abiotic stresses that are compounded by climate change (Raupach et al., 2007; Hansen et al., 2010). Both temperature and atmospheric CO_2_ are known to induce dramatic changes in plant secondary metabolites (Levine et al., 2008; Ramakrishna and Ravishankar, 2011). For example, the production of some biogenic volatile organic compounds (BVOCs) are enhanced at high temperatures (Edreva et al., 2008; Loreto and Schnitzler, 2010; Ramakrishna and Ravishankar, 2011) and the biosynthesis of plant phenolic compounds is generally enhanced by elevated CO_2_ (Lambers, 1993), although this response is often species specific (Hartley et al., 2000). Moreover, up regulation of secondary metabolism and can in turn form part of a plant’s protection strategy against short-term environmental stresses (Ramakrishna and Ravishankar, 2011).

Elevated emissions of the BVOC isoprene, synthesized though secondary metabolism *via* the non-mevalonate pathway (MEP) is commonly associated with short-term heat shock in plants (Li and Sharkey, 2013). Although the mode of action of isoprene is not fully understood, it appears to supress the negative physiological effects of extreme heat (Singsaas, 2000), presumably through its antioxidant properties protecting plant membranes (Siwko et al., 2007). Plants that emit isoprene are almost always fast growing, woody species (e.g., *Salix*) that are not subject to long periods of abiotic stresses such as extreme temperature, and thus, there is an advantage to mitigating such short-term heat shocks through secondary metabolic processes (Dani et al., 2014; Loreto and Fineschi, 2015). Isoprene is not only an important hydrocarbon in terms of plant protection against abiotic stress, but also because of its wider impact on atmospheric chemistry. The reaction of isoprene with the hydroxyl radical (· OH) for example, can influence the formation of tropospheric ozone (O_3_) (Sharkey et al., 2008) while the oxidation products of isoprene can also form secondary organic aerosols (Scott et al., 2014), which then contribute to cloud formation and its subsequent albedo effect. While capable of reducing the negative effects of high temperatures, isoprene is energetically expensive, with emissions regularly using over 50% of the carbon fixed by photosynthesis in leaves (Velikova, 2008). This release can even exceed the net amount of carbon fixed by the plant, depleting stored carbon reserves and the decoupling of isoprene emission from the prevailing rate of photosynthesis (Brilli et al., 2007; Loreto and Fineschi, 2015). As a consequence, isoprene emissions are tightly regulated *in planta* (Singsaas et al., 1997; Peñuelas et al., 2005; Velikova and Loreto, 2005; Sasaki et al., 2007) with any up regulation in the biosynthesis likely to restructure plant carbon partitioning, impacting secondary metabolic processes that are not associated with BVOC synthesis as carbon becomes more limited.

In contrast, more recent reports, including Free Air Carbon Enrichment (FACE) experiments (Sharkey and Monson, 2014) have revealed that elevated CO_2_ can supress isoprene emission under ambient temperature conditions (Possell and Hewitt, 2011). On face value, this seems counter intuitive given the high carbon cost of isoprene biosynthesis: previously it has been suggested that synthesis of secondary metabolites at elevated CO_2_ concentrations is a result of excess sucrose during protein synthesis that acts as an “energy overflow” (Lambers, 1993). However, although CO_2_ is a resource (Kainulainen et al., 1998; Mattson et al., 2005; Veteli et al., 2007), it can also be considered a stress. For example, high CO_2_ concentrations can cause dramatic, negative effects on plant physiology and profoundly affect both primary and secondary metabolism (Levine et al., 2008; Kaplan et al., 2012). These observations then potentially explain the observed suppression of isoprene emission by super-ambient CO_2_, at least at more ambient temperatures.

While the independent effects of CO_2_ and extreme temperature on the dynamics of isoprene biosynthesis have been at least partially resolved, no study has investigated the interaction of these two stressors in terms of secondary metabolism, including isoprene biosynthesis. Consequently, a mechanistic understanding of isoprene emissions is urgently needed (Wahid et al., 2012).

In order to understand the environmental regulation of isoprene production mechanistically, we urgently need to: 1) understand the impact of potential future climate change on plant secondary metabolism; 2) better understand how the MEP pathway is influenced by extreme temperature and elevated CO_2_; and 3) resolve how the interaction between these environmental factors may trade-off against one another at the metabolic level. We hypothesize that in times of elevated heat and CO_2_ concentration, the thermotolerance response of isoprene biosynthesis will not be suppressed by super-ambient CO_2_ levels, and that there is more likely to be a cancelling-out effect from the interaction of the two abiotic stresses.

In this paper, we report for the first time both the independent and combined effects of temperature and CO_2_ on the production and flux of plant secondary metabolism with a focus the flavonoids and isoprene biosynthesis using *Salix* spp. (Terra Nova [(*S. viminalis*×*Salix triandra*)×*Salix miyabeana*]). We use an untargeted metabolite fingerprint analysis to identify the pathways involved in isoprene biosynthesis, and those that are impacted on *via* the allocation of resources. Following this, a targeted metabolomics approach using nanospray tandem mass spectrometry was used to identify and confirm compounds involved in the regulation of these pathways.

## Materials and Methods

### Experiment Setup and Plant Growth

Willow cuttings of the short rotation coppice *Salix* spp. hybrid “Terra Nova” (‘LA940140 [*S. viminalis*×*Salix triandra*] × *Salix miyabeana*]) were rooted and grown in John Innes number three compost. The cuttings were grown for 10 weeks in a controlled environment chamber (Conviron BDR16, Canada) at ambient CO_2_ and day/night temperature of 20°C/15° C. Four plants for each heat shock (Hs)/elevated CO_2_ (eCO_2_) combination (16 plants in total) were selected at random and moved to acclimate for 2 weeks to either ambient or eCO_2_ treatment in the experimental chamber (Conviron BDR16, Canada), prior to the application of the Hs treatment. This experiment was repeated for replication for GC-FID analysis (32 plants in total) (see **Table 1** for treatment descriptions and **Figure S1** for flowchart of sampling strategy/replication).

**Table 1:** Environmental parameters and time periods for treatment application in controlled environment chamber (Control = ambient temperature (20°C) and ambient CO_2_ (∼400 ppm), Hs = elevated temperature (40°C), eCO_2_ = elevated CO_2_ (800 ppm).

Treatment	Parameter values		Time period	
	Temperature	CO_2_	Temperature	CO_2_
Hs*eCO_2_	40°C	800ppm	8 hours	constant
eCO_2_	20 °C	800ppm	8 hours	constant
Hs	40°C	ambient (∼400ppm)	8 hours	constant
Control	20 °C	ambient (∼400ppm)	8 hours	constant

### Air Entrainment and BVOC Collection

Entrainment equipment was sterilized 24 h before each air entrainment. Four Porapak Q adsorbent tubes (70-mm length, quartz filter) (Alltech Associates, Lancashire, UK) were eluted with 2 ml of hexane solvent and were heated at 120°C overnight to remove any contaminants. While in the drying oven, charcoal filtered air was driven through the glass tubes at 700 ml min^−1^ to facilitate contaminant removal. Four polyethylene terephthalate (PET) entrainment bags and aluminium foil (for wrapping the sterilized Porapak Q tubes) were sterilized in the drying oven overnight to remove any contaminants.

The four plants were encased individually in the PET bags. The PTFE tubing (entrainment kit) and PET bag was secured using plastic coated wire, without causing damage to the plant’s stem. Charcoal filtered air was pumped through each bag at a rate of 700 ml min^−1^ by a KNF (Switzerland) vacuum pump (Model number NMP850KNDC) and was drawn out through an outlet containing the Porapak Q tube attached to a PTFE tube at a rate of 400 ml min^−1^, which was secured in the bag using plastic coated wire. Air entrainment of the four plants began at the start of the photoperiod and ran for 6 h. At the end of the 6-h period, the Porapak Q tubes were removed from the outlet and were individually wrapped in sterilized aluminium foil. This process was repeated for each of the treatments (16 plants), and then, the experiment was repeated (32 plants, see **Figure S1** for details).

### Leaf Harvest and Metabolite Extraction

From each of the four plants following the first set of entrainments (16 plants), two leaves were selected (32 samples). A 1-cm diameter leaf discs was removed from each leaf, frozen in liquid nitrogen and stored at −80°C. Each leaf sample was freeze dried in preparation for metabolite extraction. Plant metabolites were extracted using a standard biphasic chloroform/methanol extraction according to Overy et al. (2005). 2 mg of each dried leaf sample was ground to a powder using a ball mill and 20 µl of MeOH/CHCl_3_/H_2_0 mixture (2:5:1) pre-chilled on ice for 15 min. The 32 sample tubes were then mixed for 10 s using a vortex mixer and were stored on ice for 5 min. The samples were mixed again and centrifuged at 14,000 rpm at 4°C for 2 min and the resulting supernatants were pre-chilled to –20°C. The leaf tissue pellets were re-extracted with 10µl of pre-chilled (−20°C) MeOH/CHCl_3_ (1:1) and were vortexed for 10 s. The samples were then stored on ice for 10 min. The samples were centrifuged at 14,000 rpm for 2 min at 4°C. The chloroform (organic) phase was separated from the aqueous phase by adding 4µl of chilled and distilled H_2_O and 20µl of CHCl_3_. To obtain the two clear phases (aqueous and organic) the samples were centrifuged at 14,000 rpm for 15 min at 4°C. The organic and aqueous phases (64 samples) were then stored at −80°C.

### Air Entrainment Sample Elution

The Porapak Q filter tubes were eluted with 1 ml of hexane solvent into 9-ml glass vials with a PTFE lid (SUPELCO Analytical, USA) and were stored at −20°C in preparation for gas chromatography (GC-FID) analysis of volatile compounds. This was completed following each entrainment.

### Gas Chromatography Flame Ionization Detection (Gc-Fid)

The eluted solutions (32 samples) were analyzed on a PerkinElmer AutoSystem XL GC with an auto-ignite FID. A ZB-5 column (30 m × 0.25i.d., 0.25-µm film thickness) was used. The injection temperature was 200°C with a 2-µl sample injection volume and N_2_ carrier gas. The oven temperature program used to analyze the samples is given in **Table S1**. To differentiate between the hexane solvent and isoprene, retention times for analytical standards of both the hexane solvent and a commercial isoprene standard (≥99%, Sigma-Aldrich, UK) were resolved. The retention times for hexane and isoprene were 2.915 and 2.398, respectively.

### Metabolite Profiling Using MALDI-MS

Both phases of the metabolite extracts were diluted 1:10 (MeOH). Both phases were analyzed on a Waters MALDI Synapt G2-MS (Waters, Hertfordshire) in negative ionization mode using a 9-Aminoacridine (5mg/ml) matrix. A 2-µl aliquot of the sample/matrix mix (1:1) was pipetted onto a 96-well target plate and allowed to dry. Samples were ionized using an orthogonal MALDI ion source with the laser deployed in a spiral pattern on each spot for 1 min. Each sample was analyzed in triplicate. The negative ionization mode was run using the same method as shown in **Table S2** with N_2_ carrier gas. As only the polar phase run in negative ionization mode showed significant differences, only this analysis is reported.

### Metabolite Identification Using Nanospray Tandem Mass Spectrometry

The polar phase of the metabolite extracts were diluted 1:10 (MeOH) and were analyzed in negative ionization mode on an Applied Biosystems/MDS Sciex QSTAR Elite Quadrupole Time-of-Flight (QTOF) (Sciex, Warrington) mass spectrometer using tandem mass spectrometry (MS/MS) with a nanospray head. As only the polar phase (negative ESI mode) showed differences in the fingerprint analysis, only the polar phase was used for compound validation. 2 µl of each standard compound [Apigenin, Cyanidin, Luteolin, Quercetin, Dimethylallyl diphosphate, Geranyl diphosphate, Isopentenyl diphosphate, and 2-C-Methyl-erythritol 4-phosphate (MEP)] (Sigma-Aldrich, Dorset) of the extracted sample (1:10 dilution, MeOH) was injected through a borosilicate capillary NanoES emitter (Thermo Scientific, Hertfordshire) with a flow rate of 2 µl/min and tandem mass spectrometry fragmentation patterns of the standard compounds and samples resolved. The instrument settings used to analyze each of the compounds of interest are given in **Table S3**. The resultant fragmentation patterns of the standards were compared to those of the samples and used for identification purposes, as validation that the compound was present in the sample was achieved through an almost identical fragmentation pattern match of the parent ion of interest (**Figures S4** and **S5** give the fragmentation patterns of Apigenin and MEP analytical standards and extracted samples respectively as an example of each of the secondary metabolic pathways of interest. All of the eight compounds of interest were identified and validated using this method).

### Data Processing and Statistical Analysis (GC-FID)

A calibration curve was created from the isoprene standard concentrations (nM), to define the linear range and quantify the amount of isoprene present in each of the headspace collections of the biological samples. Each calibration was calculated using the area of the peak for each analytical standard, and concentration of isoprene, expressed as nanomoles. A two-way ANOVA (Minitab version 17) was used to determine whether there was a difference in total isoprene in each sample in relation to the treatments.

### Metabolite Data Processing and Statistical Analysis

The metabolite extract spectral data were centroided and converted into text file lists of accurate masses and corresponding intensity readings to be transferred to a Microsoft Excel spreadsheet using an in-house Visual Basic macro (Overy et al., 2005). The triplicate runs of each sample were combined to calculate their mean mass, perform noise reduction, and bin the data to form the metabolite profile for each sample (Overy et al., 2005). Principal Component Analysis (PCA) using the SIMCA (Umetrics) statistical package was conducted to visualize any differences in the metabolite data results (**Figure S2**). Supervised, multivariate analysis was conducted using orthogonal partial least squares discriminant analysis (OPLS-DA) again using SIMCA (see Ringńer, 2008 for full description of discriminant analysis), to identify distinct mass bins (see Jandrić et al., 2015) that are determined by the correlation between a principal component (e.g., a *m/z* value) to the original variable (e.g., temperature) and thus account for the differences in metabolites between the different treatments. A table of the six most discriminatory bins for each treatment was compiled using online metabolite databases to putatively identify the detected masses for each bin. The putative identities of these accurate masses (monoisotopic) were resolved using the Metlin (https://metlin.scripps.edu) and Kegg (http://www.genome.jp/kegg/pathway.html) databases (see **Table S4**). The putative identities were assigned to accurate masses if the error margin of the respective *m/z* values were less than 40 ppm (De la Fuenta et al., 2017).

Significant Differences Between Treatment (Eco_2_ and Hs) for the Percentage Ion Counts of the Distinct Mass Bins Identified Above Were Resolved by Two-Way ANOVA) Using the Minitab^®^ Version 17 (Minitab Inc, 2010) Software Package and Pairwise Comparisons Resolved by *Post Hoc* Tukey HSD for Each Mass Bin. the Two Leaf Samples From Each Biological Replicate Were Pooled to Give a Per Plant Response (See **Figure S1**).

## Results

### Isoprene Concentrations in Response to Treatment

**Figure 1:**
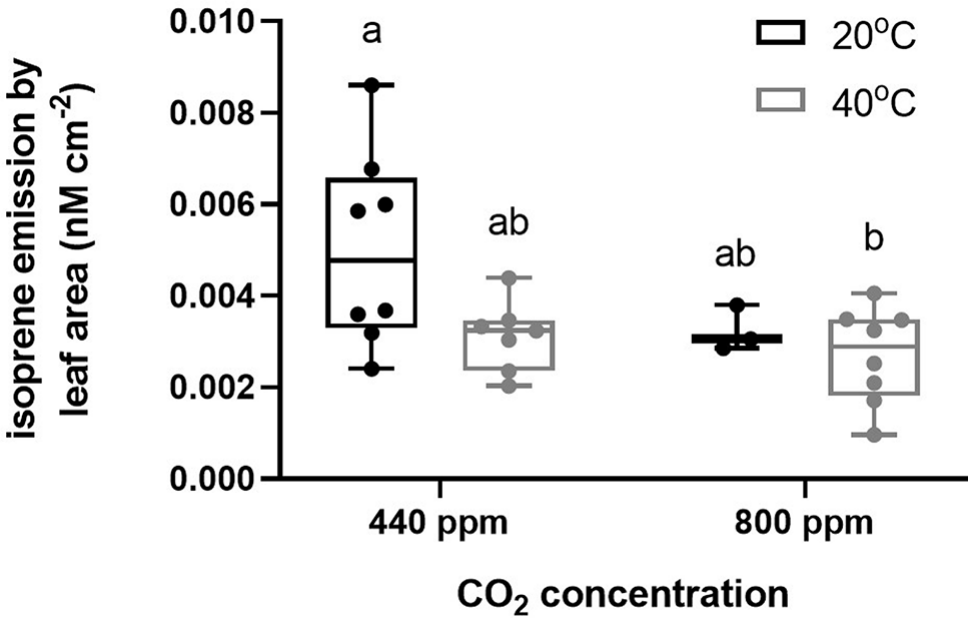
Isoprene emission measurements by leaf area (nM cm^−2^) collected from air entrainment and analysed by GC-FID according to the CO_2_ and temperature treatments (Hs, eCO_2_, Hs* eCO_2_, and control). Each data point represents one entrainment collection from each plant, and each box represents each treatment. Bars represent minimum and maximum values of the isoprene emission.

In contrast to our hypothesis, leaf and stem isoprene emissions did not significantly respond to Hs although the variance was high and the highest individual isoprene emissions of any treatment were recorded at ambient CO_2_ under elevated temperature conditions (**Figure 1**, **Table S5**). In contrast, eCO_2_ resulted in significantly lower isoprene emissions than those recorded at ambient temperature (**Figure 1**, **Table S5**) and there was no significant interaction between atmospheric CO_2_ and temperature treatments (**Table S5**).

### Temperature and CO_2_ Treatments Show Metabolite Variation in *Salix Spp*.

**Figure 2:**
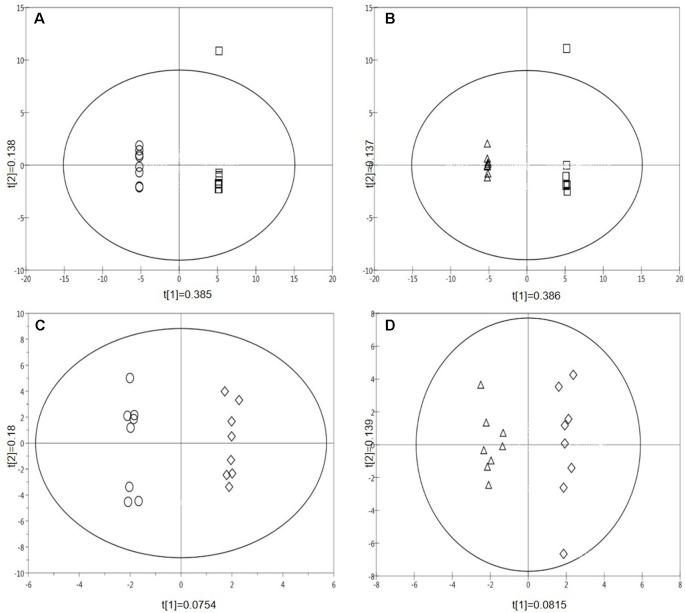
Supervised multivariate OPLS-DA plots of pair-wise comparisons of the metabolite profiles of treatments for all leaf samples (Data points from MALDI-ToF analysis, polar phase, negative ionisation mode, 32 samples). **(A)** Hs vs. control; **(B)** eCO_2_ vs. control; **(C)** Hs* eCO_2_ vs eCO_2_; **(D)** Hs*eCO_2_ vs Hs. (Circle=Hs, square=control, triangle=eCO_2_, diamond=Hs*eCO_2_).

A supervised pairwise OPLS-DA analysis revealed differences in the metabolic fingerprints of the four treatments (ambient temperature and ambient CO_2_; ambient temperature and eCO_2_; Hs and ambient CO_2_; Hs and eCO_2,_ see **Table 1**) (**Figures 2A–D**). Plots (A) and (B) show a separation between both the Hs treatment and the control, and the eCO_2_ treatment and the control, respectively in terms of metabolic fingerprints. As well as comparing Hs and eCO_2_ treatments to the control, the treatments have been compared to each other; eCO_2_ and Hs compared to eCO_2_ alone (**Figure 2C**) and eCO_2_ and Hs compared to Hs alone (**Figure 2D**). Both plots revealed that the effect of eCO_2_ and Hs induce a response in terms of plant metabolism. **Figures 2A–D** demonstrate not only the independent effects of the abiotic stresses, but also their interactive effects in terms of influencing plant secondary metabolism. The loadings plots for each OPLS-DA analysis (**Figure S3**, **Table S6** for statistics) were resolved in order to identify the mass bins associated with the treatments. The identified mass bins from the multivariate analysis were interrogated to assess the differences in abundance of individual secondary metabolites between the treatments and the control group, with the most frequently represented chemical groups that were putatively identified from the Metlin and KEGG databases being flavones, glucosides, flavonoids, and phenylpropanoids (see **Table S4** for full list). The treatment-specific responses of these compound classes are explored in detail in the following sections.

### Discriminatory Mass Bin Identification Shows Down Regulation of Flavonoids in Response to Both CO_2_ and Temperature

**Figure 3:**
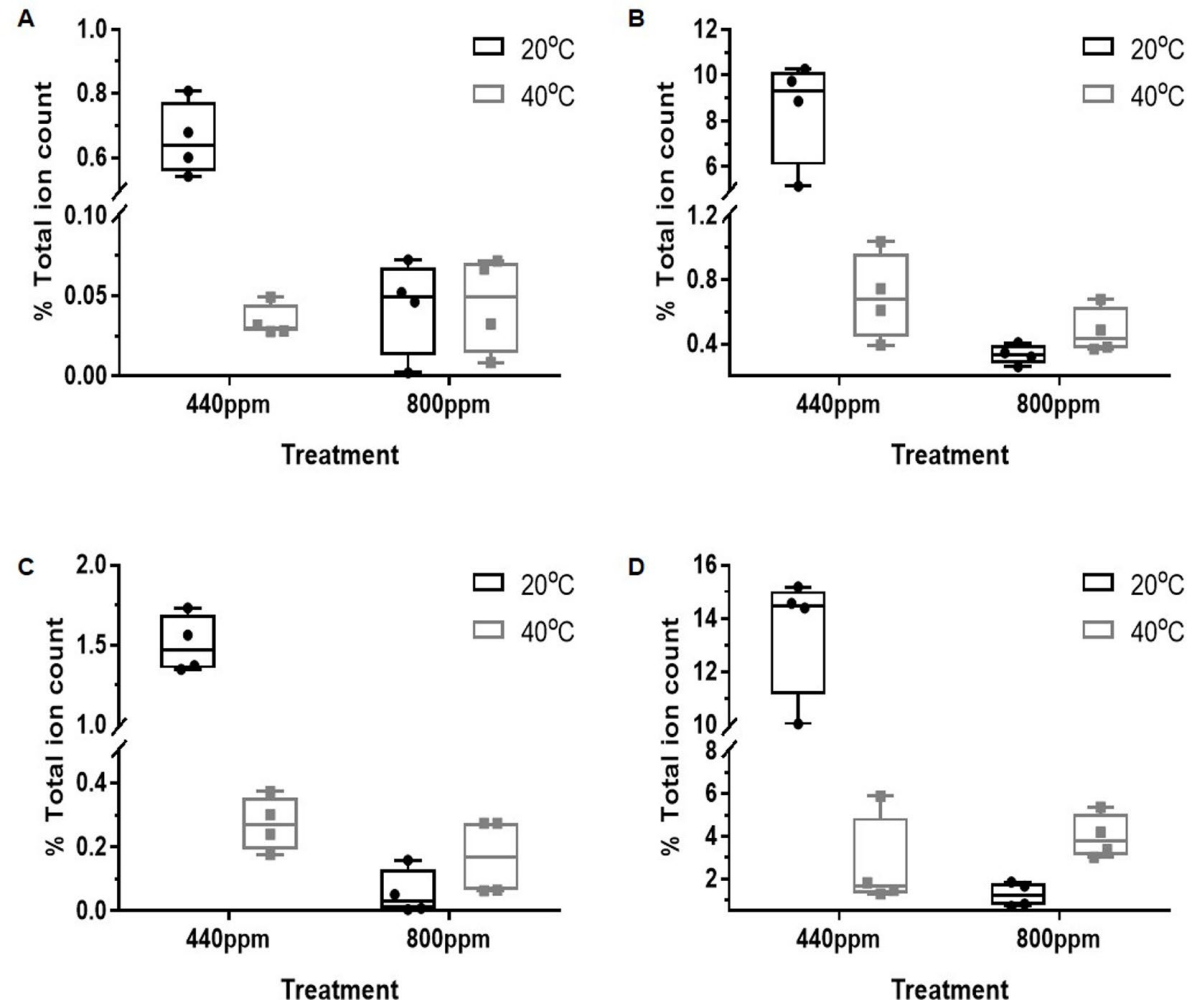
Total % ion count of discriminatory mass bins (m/z) from MALDI-ToF analysis of polar phase, negative ionisation mode. The mass bins represented are defined as containing a number of putatively identified secondary metabolites found in the Flavonoid biosynthesis pathway including the compounds: Apigenin, Cyanidin, Luteolin, and Quercetin. The graphs show less abundance of the %TIC of the secondary metabolites within the bins under the CO_2_ and temperature treatments when compared to the control treatment. **(A)** (bin 269); **(B)** (bin 285); **(C)** (bin 301); and **(D)** (bin 447). Each point represents a pooled replicate per plant across the four treatments. Bars represent minimum and maximum values.

Average intensities of the percentage total ion count for the discriminatory mass bins associated with the flavonoids (*m/z* bins 269, 285, 301, and 447, corresponding to putatively identified flavonoids Apigenin, Cyanidin, Quercetin, and Luteolin, respectively) are shown in **Figure 3**. At ambient CO_2_, all of the mass bins associated with the flavonoids were supressed by Hs (**Figure 3**, **Table S4**). In contrast, the relative abundance of the total ion counts in *m/z* bin 269 (A) was not affected by temperature at eCO_2_, while the percentage ion counts in *m/z* bins 285 (B), 301 (C), and 447 (D) increased in response to temperature at eCO_2_. Overall, however, the relative abundance of the percentage total ion counts of all of these mass bins was lower in the eCO_2_ treatments when compared with the control treatment.

### Discriminatory Mass Bin Identification Shows Up Regulation of Shikimate Pathway Metabolites in Response to Both CO_2_ and Temperature

**Figure 4:**
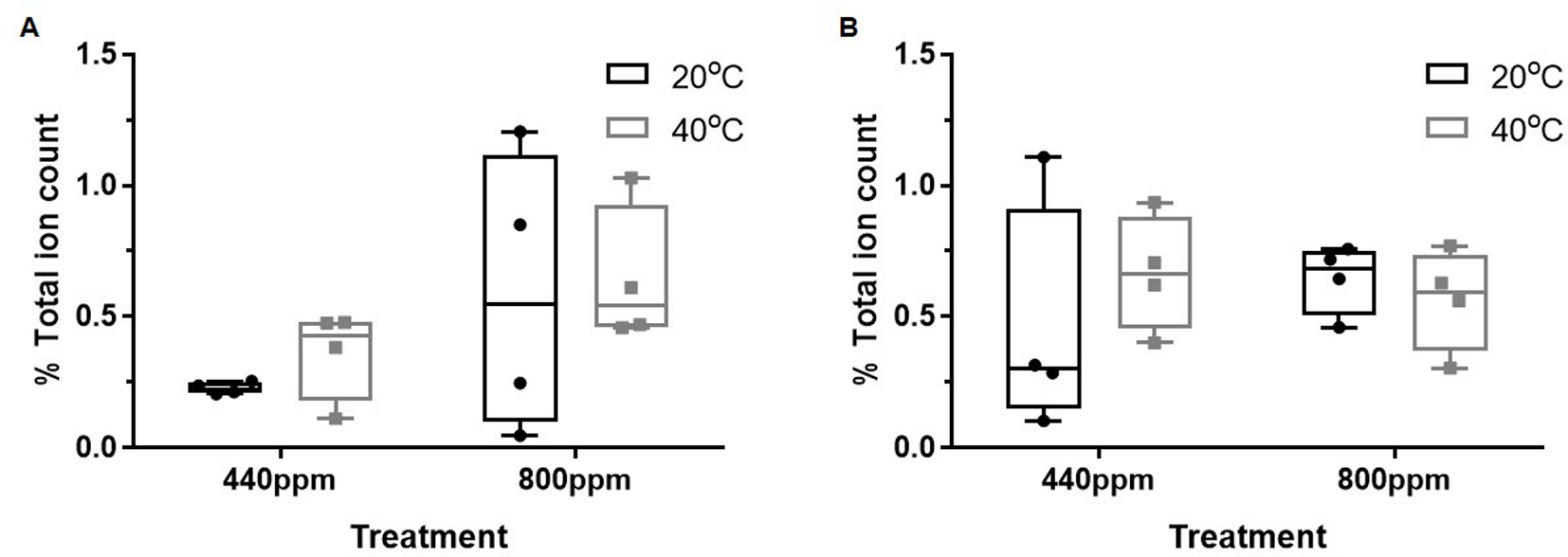
Total % ion count of discriminatory mass bins (m/z) from MALDI-ToF analysis of polar phase, negative ionisation mode. The mass bins represented are defined as containing putatively identified secondary metabolites involved in glycolysis and the shikimate pathway that show greater abundance of %TIC of the secondary metabolites within the bins under the CO_2_ and temperature treatments when compared to the control treatment. **(A)** (bin 385) and **(B)** (bin 365). Each point represents a pooled replicate per plant across the four treatments. Bars represent minimum and maximum values.

In contrast to the suppression of the flavonoids, (**Figures 3**), relative abundance (percentage total ion count) of the chemical groups putatively identified as part of glycolysis and the shikimate pathways were increased relative to the control treatment (**Figures 4A, B**). At both ambient and eCO_2_, the *m/z* bins (and corresponding secondary metabolites) associated with the shikimate pathway [385: Isosamidin (A)) and glycolysis (365: Salicin 6-phosphate (B)] were increased in relative abundance when compared with the control treatment in the *m/z* bin 365 attributed to glycolysis.

### Response of Biochemical Precursors of Isoprene in Non-Mevalonate Pathway (MEP) and Flavonoid Metabolic Pathway in Response to Both CO_2_ and Temperature

**Figure 5:**
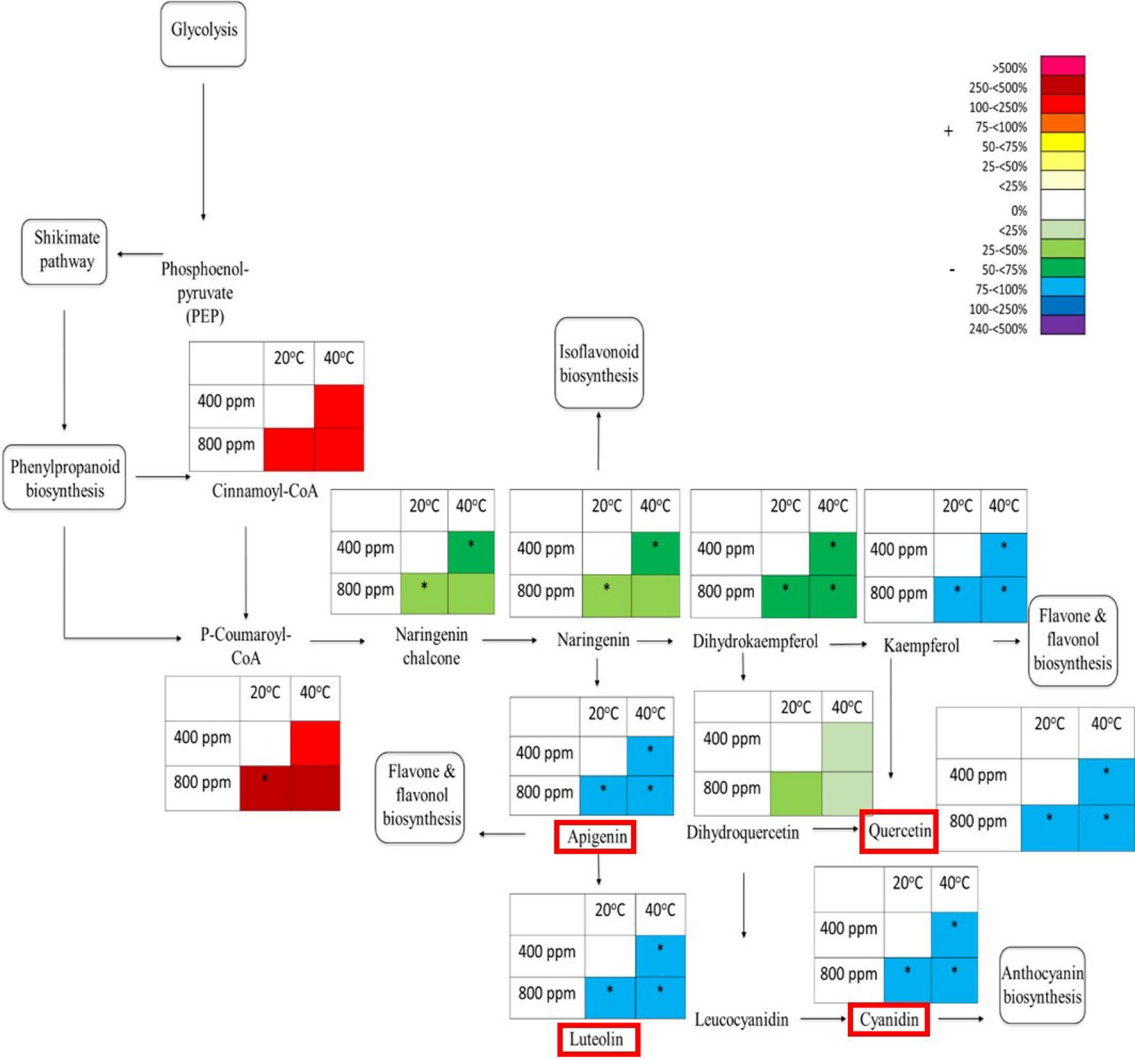
Schematic pathway diagram of glycolytic, shikimate, phenylpropanoid and flavonoid biosynthesis. The tables show abundance of the metabolites calculated from the % total ion count for data from MALDI-ToF analysis (polar phase analysed in negative ionisation mode). * indicates statistically significant changes in % differences of the total ion count relative to treatment. The % bar scale shows increase or decrease in abundance of %TIC. Red boxes around specific secondary metabolites indicate identification using tandem MS against an analytical standard. Each point in the table for each metabolite represents a pooled replicate per plant across the four treatments.

Given that the flavonoids (**Figure 3**) tend to be supressed by abiotic stresses, and in contrast the *m/z* bins 385 (putatively identified as Isosamidin and involved in glycolysis) and 365 (putatively identified as Salicin 6-phosphate and part of the shikimic acid pathway, **Figure 4**) are enhanced by abiotic stresses, we mapped the responses of the *m/z* bins associated with the metabolite intermediates of the glycolytic and shikimic acid pathways to eCO_2_ and Hs. Four metabolites within the flavonoid metabolic pathway were selected to be quantitatively identified using tandem mass spectrometry (see **Table S3** for list of compounds) to be compared against known analytical standard fragmentation patterns (**Figure S4** gives an example of Apigenin analytical standard and plant tissue sample fragmentation patterns). Following confirmation through ms/ms fragmentation, the relative abundance of the ions associated with the four confirmed intermediates of the flavonoid biosynthetic pathway were mapped along with other putatively identified intermediates within the pathway (**Figure 5**). The pathway map shows the effect on the percentage total ion counts of the *m/z* bins and their associated metabolites by the eCO_2_ and Hs treatments. At the beginning of the flavonoid pathway, both metabolite intermediates Cinnamoyl-CoA and P-coumaroyl-CoA show an increase in abundance at both eCO_2_ and Hs when compared with the control treatment. At eCO_2_, P-coumaroyl-CoA showed a significant increase of 250%–500% when compared to the control treatment. Cinnamoyl-CoA had a less marked increase (75%–100%), but still showed an increase in metabolite abundance when compared to the control at both eCO_2_ and Hs. However, downstream of these intermediates, there is a suppression of metabolite intermediates in the flavonoid pathway. Naringenin chalcone showed a significant decrease of 25%–75% in times of eCO_2_ at ambient temperature, and also when subject to Hs at ambient CO_2_ concentration. There was also a trend that during the combined eCO_2_ and Hs treatment, this metabolite was suppressed in contrast to the control treatment. The following intermediate in the pathway, Naringenin also showed the same significant decrease as Naringenin chalcone. However, Dihyrdoquercetin showed less of a decrease in metabolite abundance at Hs, and combined eCO_2_ and Hs (− < 25%). This intermediate did still show a greater decrease in abundance at eCO_2_ when compared to the control treatment (−25%–50%), following the same trend as Naringenin chalcone, and Naringenin. Kaempferol showed the same significant decrease in metabolite abundance as the four intermediates that were confirmed using tandem MS (Apigenin, Quercetin, Cyanidin, and Luteolin). In all treatments (both eCO_2_ and Hs), there was a significant decrease of 75%–100% when compared the to the control treatment, and greater than that of the upstream metabolites that have been discussed.

**Figure 6:**
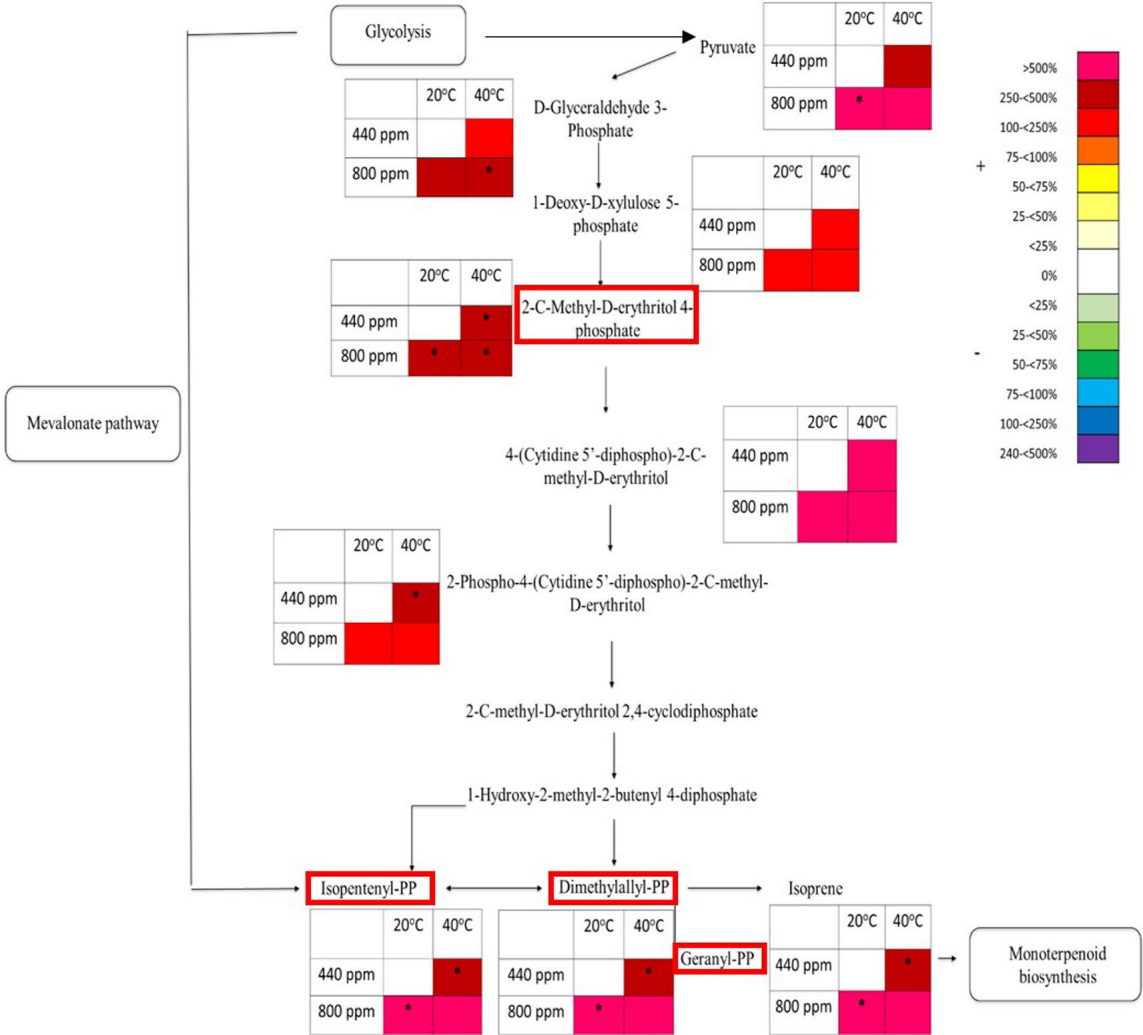
Schematic pathway diagram of glycolytic, mevalonate, non-mevalonate and monoterpenoid biosynthesis. The tables show abundance of the metabolites calculated from the % total ion count for data from MALDI-ToF analysis (polar phase analysed in negative ionisation mode). * indicates statistically significant changes in % differences of the total ion count relative to treatment. The % bar scale shows increase or decrease in abundance of %TIC. Red boxes around specific secondary metabolites indicate identification using tandem MS against an analytical standard. Each point in the table for each metabolite represents a pooled replicate per plant across the four treatments.

Four metabolite precursors to isoprene production in the isoprene biosynthesis pathway were also selected to be quantitatively identified using tandem mass spectrometry (see **Table S3** for list of compounds) to be compared against known analytical standard fragmentation patterns (**Figure S5** gives an example of 2-C-methyl-D-erythritol 4-phosphate (MEP) analytical standard and plant tissue sample fragmentation patterns). In contrast to the suppression that has been seen in the flavonoid pathway map, the intermediates within the MEP pathway map (**Figure 6**) showed an increase in the abundance of percentage total ion counts for each of the metabolites. Pyruvate, a precursor to the MEP pathway showed an increase in abundance at both Hs and eCO_2_ of 250%–500%. There was a significant increase in metabolite abundance at eCO_2_ when compared to the control treatment. D-glyceraldehyde 3-phosphate, 1-deoxy-D-xlyulose 5-phosphate, 2-C-methyl-D-erythritol 4-phosphate, 4-(cytidine 5’-diphospho)-2-C-methyl-D-erythritol, and 2-phospho-4-(cytidine 5’-diphospho)-2-C-methyl-D-erythritol all demonstrated a marked increase (ranging from 100% to 500%) in metabolite abundance in all eCO_2_ and Hs treatments when compared to the control treatment. 2-C-methyl-D-erythritol 4-phosphate in particular shows a significant increase in each of the treatments, and 2-phospho-4-(cytidine 5’-diphospho)-2-C-methyl-D-erythritol has a significant increase at Hs. A number of metabolites (2-C-methyl-D-erythritol 2,4-cyclodiphosphate, and 1-hydroxy-2-methyl-2-butenyl 4-diphosphate) could not be identified by percentage total ion count from their associated *m/z* bin and have therefore not been included in the MEP pathway map. Isopentenyl-PP, Dimethylallyl-PP, and Geranyl-PP also showed the same increase in metabolite abundance of 250%–500% at Hs at ambient CO_2_, and >500% at both eCO_2_ at ambient temperature and the combined abiotic stresses (Hs*eCO_2_). There was no significant interactive effect of the Hs and eCO_2_ treatments, but there was a significant increase in metabolite abundance in the independent treatments (Hs or eCO_2_).

## Discussion

The BVOC isoprene is produced in response to heat stress and its biosynthesis is known to be supressed under conditions of eCO_2_. However, no study has investigated the interaction of these two stressors in terms of secondary metabolism, including isoprene biosynthesis. We hypothesized that in times of Hs and eCO_2_ concentration, the thermotolerance response of isoprene biosynthesis will not be suppressed by super-ambient CO_2_ levels, and that there is more likely to be a levelling (cancelling-out) effect from the interaction of the two abiotic stresses. These predictions, however, were not fully supported by our experiments.

### Response of Isoprene Biosynthesis and Emission to Elevated Temperature and CO_2_

Short-term heat shocks, and increased atmospheric CO_2_ concentration are known to induce shifts in plant secondary metabolism. Previous studies have shown that isoprene emissions increase exponentially to elevated temperature until *circa* 40°C (Velikova, 2008), with no apparent damage to the plant’s photosystem (Sharkey, 2005), after which point emissions sharply decline (Velikova, 2008). This suggests that there is some benefit to the plant in allocating carbon resources to the biosynthesis of isoprene through thermotolerance (Singsaas et al., 1997; Peñuelas et al., 2005; Velikova and Loreto, 2005; Sasaki et al., 2007).

Although isoprene biosynthesis is energetically expensive, its antioxidant nature and ability to protect the cell membrane indicates that these physiological benefits far outweigh their energetic cost (Wahid et al., 2012). That said, given the relative energetic costs of producing BVOCs, any up regulation in the biosynthesis of isoprene is likely to restructure plant carbon partitioning and effect secondary metabolism not associated with BVOC synthesis as carbon becomes more limited. Based on first principles, however, it could be assumed that eCO_2_ would, to some extent, mitigate the extreme carbon demand placed on the plant by the biosynthesis of isoprene and provide substrate for the production of other secondary metabolites in times of abiotic stress. It has been hypothesized previously that under times of eCO_2_, there will be an increase in the production of secondary metabolites due to an “energy overflow” from the production of proteins (Lambers, 1993). However, the results of this study have demonstrated the opposite in relation to secondary metabolic processes. As discussed in more detail in the following sections, secondary metabolic production is inhibited during periods of Hs and eCO_2_ stress as substrate is being diverted away from pathways such as the flavonoids towards intermediates in the MEP pathway. Lambers (1993) has proposed an alternative model that provides an explanation for the restructuring of plant carbon partitioning that has been seen in this study. The “*amino acid diversion model of plant secondary metabolism*” proposes that there is no direct effect of increased secondary metabolite production in times of eCO_2_ concentration. Both amino acid synthesis and the production of phenolic compounds share the shikimate pathway (Lambers, 1993). This model offers an explanation as to why in times of stress, we see flavonoid pathway suppression but an increase in abundance of metabolites in the shikimate pathway.

### Short-Term Heat Shock Effects on Secondary Metabolism

The results of the pathway analysis are consistent with previous studies at high temperature (Behnke et al., 2010). A down-regulation of phenolic biosynthesis occurs in poplar trees subjected to Hs. The results are also consistent with the studies of Paajanen et al. (2011), and Veteli et al. (2002; 2007), where an overall reduction in the concentration of phenolic compounds that form part of the flavonoid and anthocyanin pathways, e.g., Quercetin and Kaempferol 7-*O*-glucoside (Treutter, 2006) occur in response to Hs.

Suppression of phenolic compounds occurs at high temperatures, most likely as a result of the degradation of both flavonoids and anthocyanins (Dela et al., 2003; Behnke et al., 2010). Isoprene has the ability to recycle free radicals and other reactive oxygen species that could potentially cause oxidative stress to the plant (Wolfertz et al., 2003). As isoprene is costly for the plant to produce, it could be that the plant is allocating resources to the production and emission of a secondary metabolite that has both antioxidant and thermotolerance properties and is therefore biochemically viable and beneficial. As both the terpenoid and flavonoid biosynthesis pathways share pyruvic acid following the conversion of glucose *via* the glycolysis pathway and citric acid cycle, it would make sense to have a “directional switch” as a result of substrate competition. This would allow the available substrate to be allocated to the more appropriate and immediate response to short-lived abiotic stress (Wahid et al., 2012). This has been observed by Loreto and Sharkey (1993) who found that short-term isoprene emission concentration increases positively and correlates with increases in adenosine triphosphate (ATP) in leaves in times of short-term heat stress.

The regulation of pyruvate, feeding directly into the MEP/DOXP pathways suggests that recently photosynthesized carbon has been transported from the Calvin-Benson cycle. Although it has been found in ^13^C labelling experiments that 77% of recently fixed carbon in heat stressed plants comes from pyruvate (Funk et al., 2004), the exact source of pyruvate is not clearly understood (Banerjee and Sharkey, 2014) and requires further interrogation.

### Elevated CO_2_ Concentration Effects on Secondary Metabolism

Plants grown at eCO_2_ concentrations exhibit less of a change to their biochemistry than those subjected to periods of extreme heat (Taub, 2010; Ramakrishna and Ravishankar, 2011). This is both demonstrated in the isoprene emissions measurements and the relative abundance of plant metabolites in the discriminant mass bins relating to glycolysis and the shikimate pathway (**Figure 4**). The intermediates in the shikimate pathways recognized in discriminant mass bin 385 demonstrates an up-regulation of shikimic acid and tryptophan in all treatments when compared to the control.

There is evidence in the literature that isoprene is synthesized from a number of different carbon sources (Funk et al., 2004), and that pyruvate and phosphoenolpyruvate (PEP) in particular could be a route into the chloroplast for isoprene biosynthesis (Banerjee and Sharkey, 2014).

It is known that super-ambient CO_2_ leads to enhanced energy flows through glycolysis and other metabolic pathways that are associated with central carbon metabolism, including pyruvate (Li and Sharkey 2013; Watanabe et al., 2014). This then has the potential to lessen the trade-off between carbon allocation to the MEP pathway and other essential secondary metabolic processes.

PEP has a significant role as an intermediate in the biosynthesis of glucose *via* the gluconeogenic pathway and citric acid cycle (TCA), as its metabolism leads to pyruvate and ATP transfer, which can then be used for the biosynthesis of phenolic compounds through the shikimate pathway and thus facilitate carbon fixation (Dizengremel et al., 2012; Trowbridge et al., 2012; Kaling et al., 2014). Li et al. (2008) have also found that in increase in CO_2_ leads to an observed increase in the diversity of plant secondary metabolites. They found that an increase in cell growth in young leaves when compared with ambient air. The results of this study have observed an increase in the relative abundance of secondary metabolites associated with the shikimate pathway and glycolysis in young leaves. The present study parallels with that of Ainsworth et al. (2006) and Li et al. (2008) in the observation of glucose, galactose, and maltose compounds increase as a response to eCO_2_. The increase in glycolysis intermediates are required for PEP formation and metabolism, therefore provide a flux of substrate into the citric acid cycle for the biosynthesis of secondary metabolites and energy production (Ainsworth et al., 2006). This adds strength to the idea that an increase in CO_2_ concentration is leading to an increase in carbon partitioning in the plant, therefore providing up-regulation in secondary metabolites. The same conclusion has been drawn by Veteli et al. (2007) that eCO_2_ concentrations are contributing to an increase in carbon-rich secondary metabolites.

Suppression of phenolic compounds occurs at high temperatures, most likely as a result of the degradation of both the flavonoids and the anthocyanins (Dela et al., 2003; Behnke et al., 2010). Isoprene has the ability to recycle free radicals and other reactive oxygen species that could potentially cause oxidative stress to the plant (Wolfertz et al., 2003). As isoprene is costly for the plant to produce, it could be that the plant is allocating resources to the production and emission of a secondary metabolite that has both antioxidant and thermotolerance properties and is therefore biochemically viable and beneficial. As both the terpenoid and flavonoid biosynthesis pathways share pyruvic acid following the conversion of glucose *via* the glycolysis pathway and citric acid cycle, it would make sense to have a “directional switch” as a result of substrate competition. This would allow the available substrate to be allocated to the more appropriate and immediate response to short-lived abiotic stress (Wahid et al., 2012). This has been observed by Loreto and Sharkey (1993) who found that short-term isoprene emission concentration increases positively and correlates with increases in adenosine triphosphate (ATP) in leaves in times of short-term heat stress.

## Conclusion

The aim of this study was to resolve the biochemical mechanisms underpinning the regulation of isoprene production by Hs and eCO_2_. This study has demonstrated the impact of both the independent, and the interactive effects of Hs and eCO_2_ upon woody plant (*Salix* spp.) secondary metabolism, and in particular, isoprene biosynthesis. The results of this study have revealed that in times of abiotic stress, plant secondary metabolism is drastically altered, with substrate being diverted to energetically expensive secondary metabolic pathways. With global temperature and atmospheric CO_2_ concentrations rising as a result of anthropogenic activity, it is imperative to understand the interactions between atmospheric processes and global vegetation, especially given that global isoprene emissions have the potential to contribute to atmospheric warming mitigation.

## Data Availability Statement

All datasets generated for this study are included in the article/Supplementary Material.

## Author Contributions

NA and DC designed the study. NA performed the research, data analysis, and wrote the first draft of the manuscript. JL, HW, GP, and DC wrote sections of the manuscript. Authors contributed to manuscript revision and approved the final version.

## Funding

This research was conducted through the Energy-Futures Doctoral Training Centre, funded by the Engineering and Physical Sciences Research Council (EPSRC). DC is supported by a Royal Society University Research Fellowship EPSRC funding: EP/G037477/1.

## Conflict of Interest

The authors declare that the research was conducted in the absence of any commercial or financial relationships that could be construed as a potential conflict of interest.
