# Supplementary material for: The Regulation of Plant Secondary Metabolism in Response to Abiotic Stress: Interactions Between Heat Shock and Elevated CO2
Source: Front Plant Sci. 2019 Nov 14;10:1463. doi: 10.3389/fpls.2019.01463 (PMC6868642; doi:10.3389/fpls.2019.01463)
Supplement: Supplementary file 1 [file Presentation_1.pptx]

## Slide 1
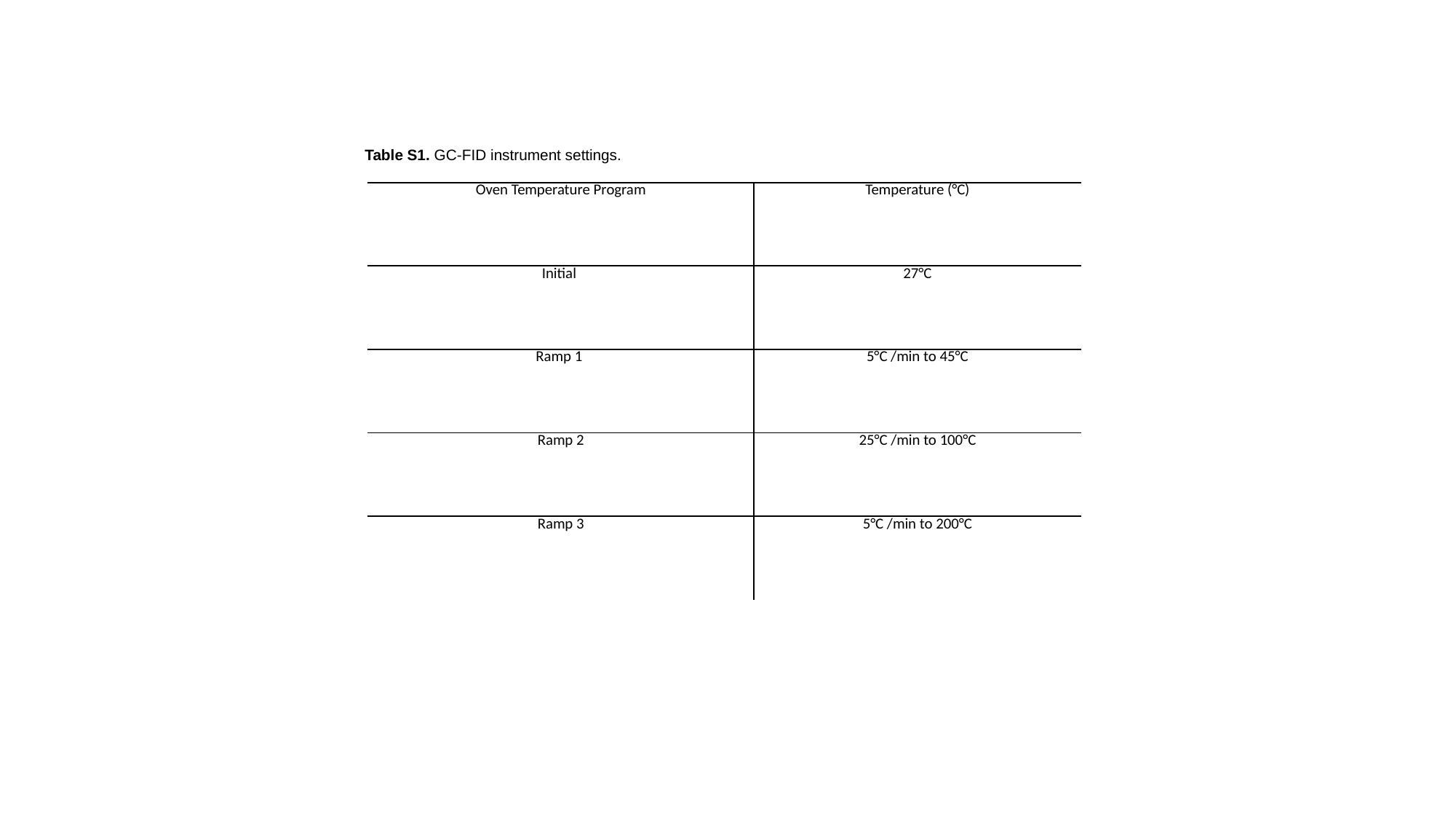

Table S1. GC-FID instrument settings.
| Oven Temperature Program | Temperature (°C) |
| --- | --- |
| Initial | 27°C |
| Ramp 1 | 5°C /min to 45°C |
| Ramp 2 | 25°C /min to 100°C |
| Ramp 3 | 5°C /min to 200°C |

## Slide 2
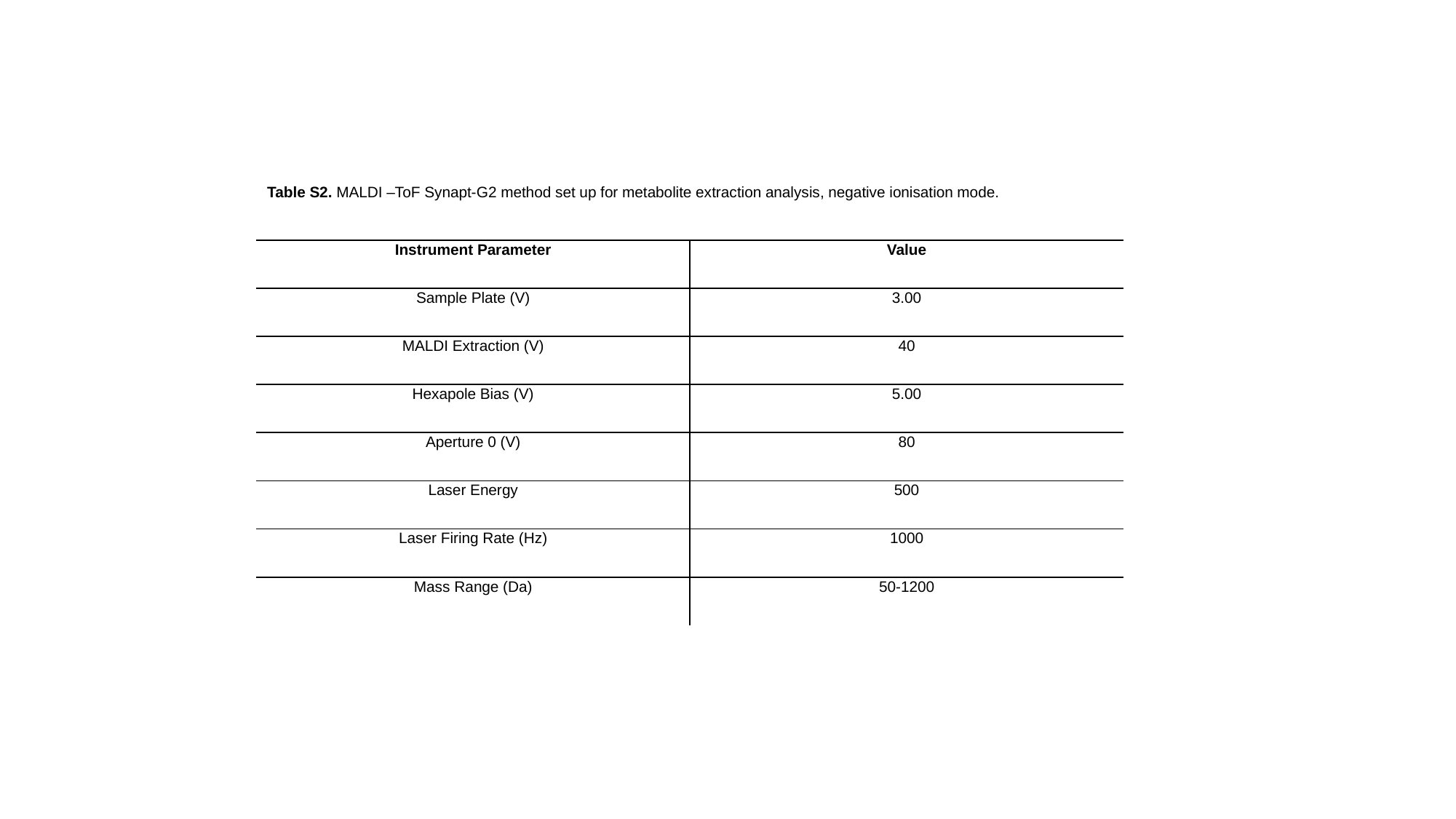

Table S2. MALDI –ToF Synapt-G2 method set up for metabolite extraction analysis, negative ionisation mode.
| Instrument Parameter | Value |
| --- | --- |
| Sample Plate (V) | 3.00 |
| MALDI Extraction (V) | 40 |
| Hexapole Bias (V) | 5.00 |
| Aperture 0 (V) | 80 |
| Laser Energy | 500 |
| Laser Firing Rate (Hz) | 1000 |
| Mass Range (Da) | 50-1200 |

## Slide 3
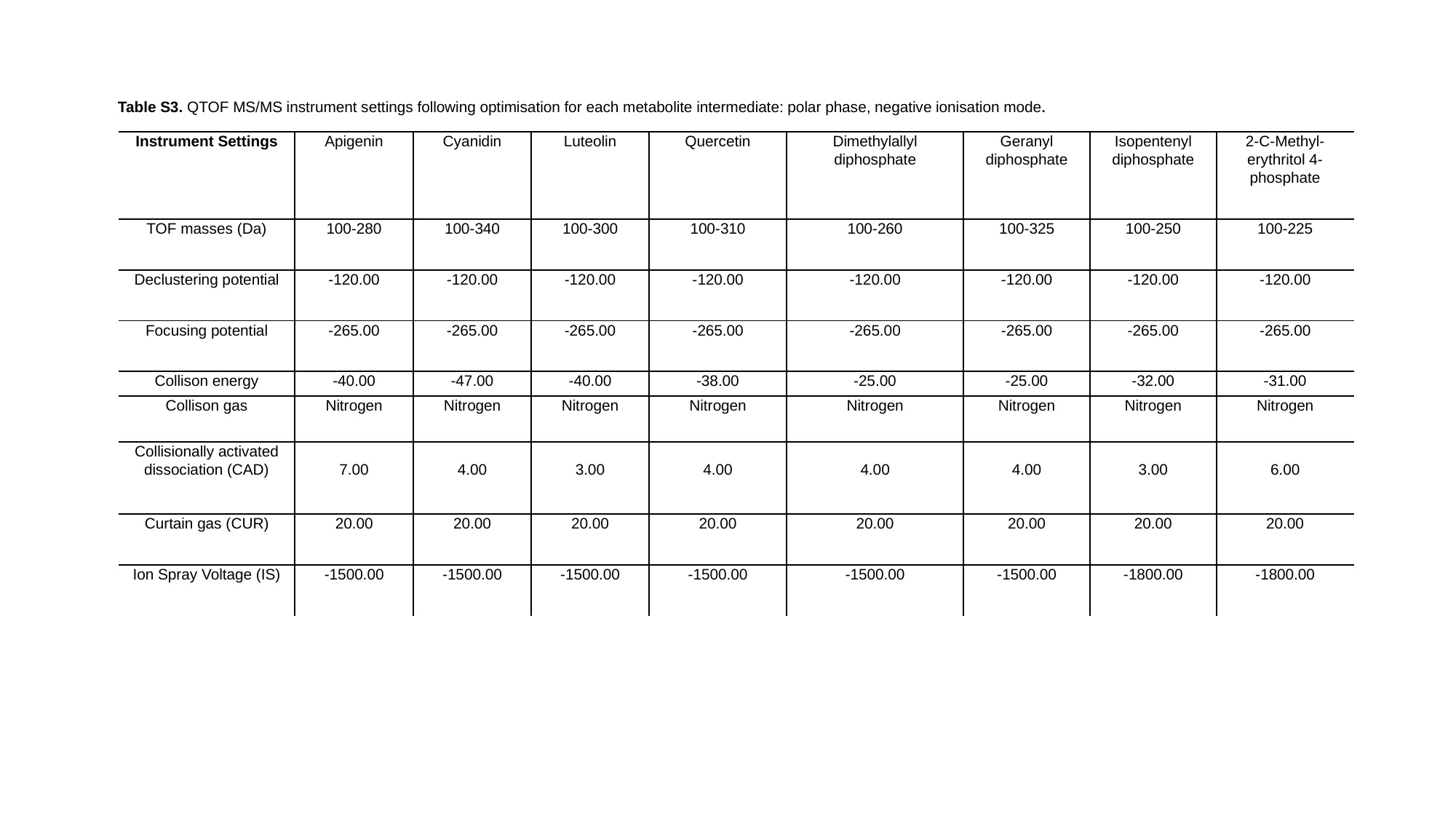

Table S3. QTOF MS/MS instrument settings following optimisation for each metabolite intermediate: polar phase, negative ionisation mode.
| Instrument Settings | Apigenin | Cyanidin | Luteolin | Quercetin | Dimethylallyl diphosphate | Geranyl diphosphate | Isopentenyl diphosphate | 2-C-Methyl-erythritol 4-phosphate |
| --- | --- | --- | --- | --- | --- | --- | --- | --- |
| TOF masses (Da) | 100-280 | 100-340 | 100-300 | 100-310 | 100-260 | 100-325 | 100-250 | 100-225 |
| Declustering potential | -120.00 | -120.00 | -120.00 | -120.00 | -120.00 | -120.00 | -120.00 | -120.00 |
| Focusing potential | -265.00 | -265.00 | -265.00 | -265.00 | -265.00 | -265.00 | -265.00 | -265.00 |
| Collison energy | -40.00 | -47.00 | -40.00 | -38.00 | -25.00 | -25.00 | -32.00 | -31.00 |
| Collison gas | Nitrogen | Nitrogen | Nitrogen | Nitrogen | Nitrogen | Nitrogen | Nitrogen | Nitrogen |
| Collisionally activated dissociation (CAD) | 7.00 | 4.00 | 3.00 | 4.00 | 4.00 | 4.00 | 3.00 | 6.00 |
| Curtain gas (CUR) | 20.00 | 20.00 | 20.00 | 20.00 | 20.00 | 20.00 | 20.00 | 20.00 |
| Ion Spray Voltage (IS) | -1500.00 | -1500.00 | -1500.00 | -1500.00 | -1500.00 | -1500.00 | -1800.00 | -1800.00 |

## Slide 4
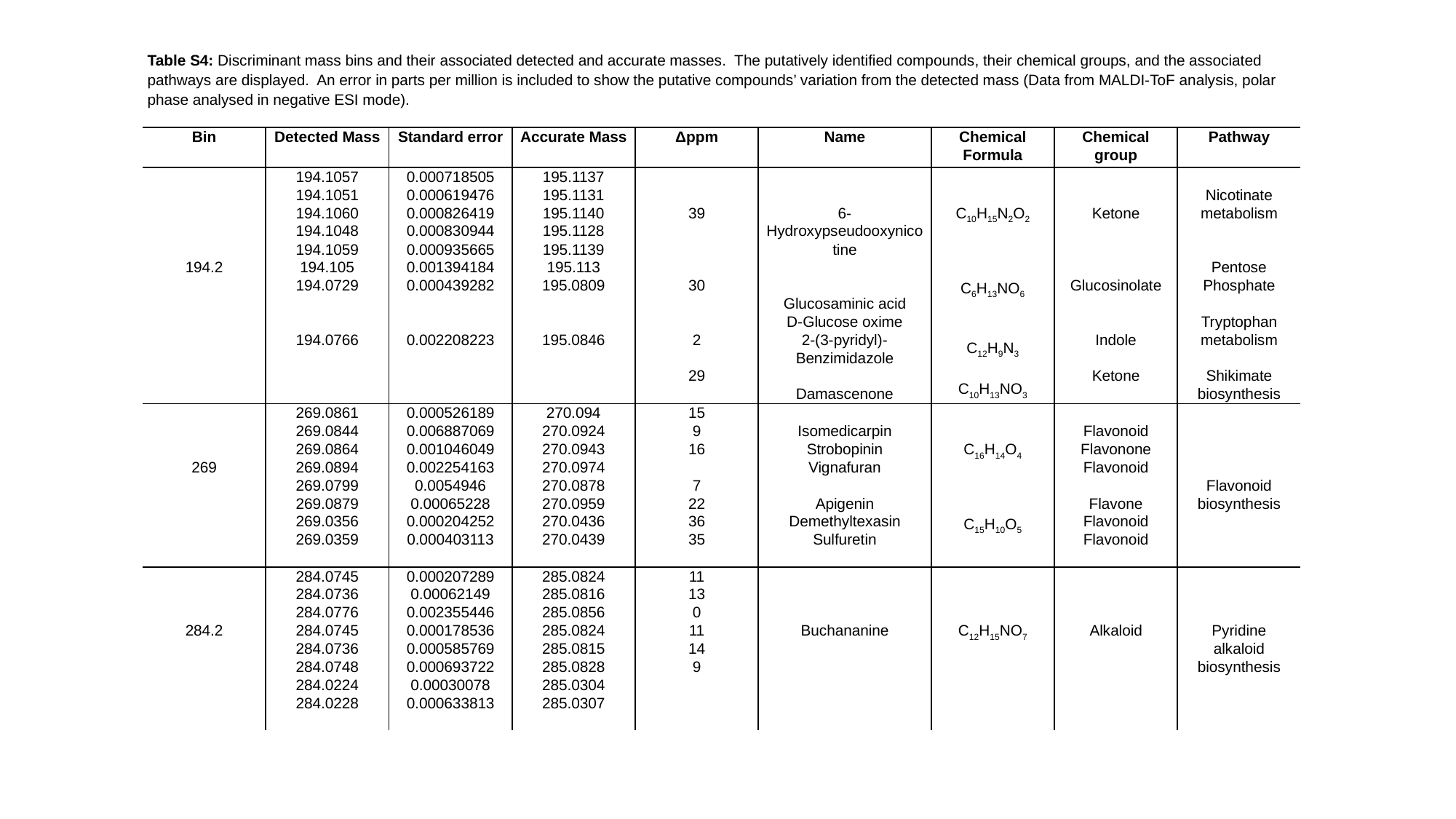

Table S4: Discriminant mass bins and their associated detected and accurate masses. The putatively identified compounds, their chemical groups, and the associated pathways are displayed. An error in parts per million is included to show the putative compounds’ variation from the detected mass (Data from MALDI-ToF analysis, polar phase analysed in negative ESI mode).
| Bin | Detected Mass | Standard error | Accurate Mass | Δppm | Name | Chemical Formula | Chemical group | Pathway |
| --- | --- | --- | --- | --- | --- | --- | --- | --- |
| 194.2 | 194.1057 194.1051 194.1060 194.1048 194.1059 194.105 194.0729     194.0766 | 0.000718505 0.000619476 0.000826419 0.000830944 0.000935665 0.001394184 0.000439282     0.002208223 | 195.1137 195.1131 195.1140 195.1128 195.1139 195.113 195.0809     195.0846 | 39       30     2   29 | 6-Hydroxypseudooxynicotine     Glucosaminic acid D-Glucose oxime 2-(3-pyridyl)- Benzimidazole   Damascenone | C10H15N2O2       C6H13NO6     C12H9N3   C10H13NO3 | Ketone       Glucosinolate     Indole   Ketone | Nicotinate metabolism     Pentose Phosphate   Tryptophan metabolism   Shikimate biosynthesis |
| 269 | 269.0861 269.0844 269.0864 269.0894 269.0799 269.0879 269.0356 269.0359 | 0.000526189 0.006887069 0.001046049 0.002254163 0.0054946 0.00065228 0.000204252 0.000403113 | 270.094 270.0924 270.0943 270.0974 270.0878 270.0959 270.0436 270.0439 | 15 9 16   7 22 36 35 | Isomedicarpin Strobopinin Vignafuran   Apigenin Demethyltexasin Sulfuretin | C16H14O4       C15H10O5 | Flavonoid Flavonone Flavonoid   Flavone Flavonoid Flavonoid | Flavonoid biosynthesis |
| 284.2 | 284.0745 284.0736 284.0776 284.0745 284.0736 284.0748 284.0224 284.0228 | 0.000207289 0.00062149 0.002355446 0.000178536 0.000585769 0.000693722 0.00030078 0.000633813 | 285.0824 285.0816 285.0856 285.0824 285.0815 285.0828 285.0304 285.0307 | 11 13 0 11 14 9 | Buchananine | C12H15NO7 | Alkaloid | Pyridine alkaloid biosynthesis |

## Slide 5
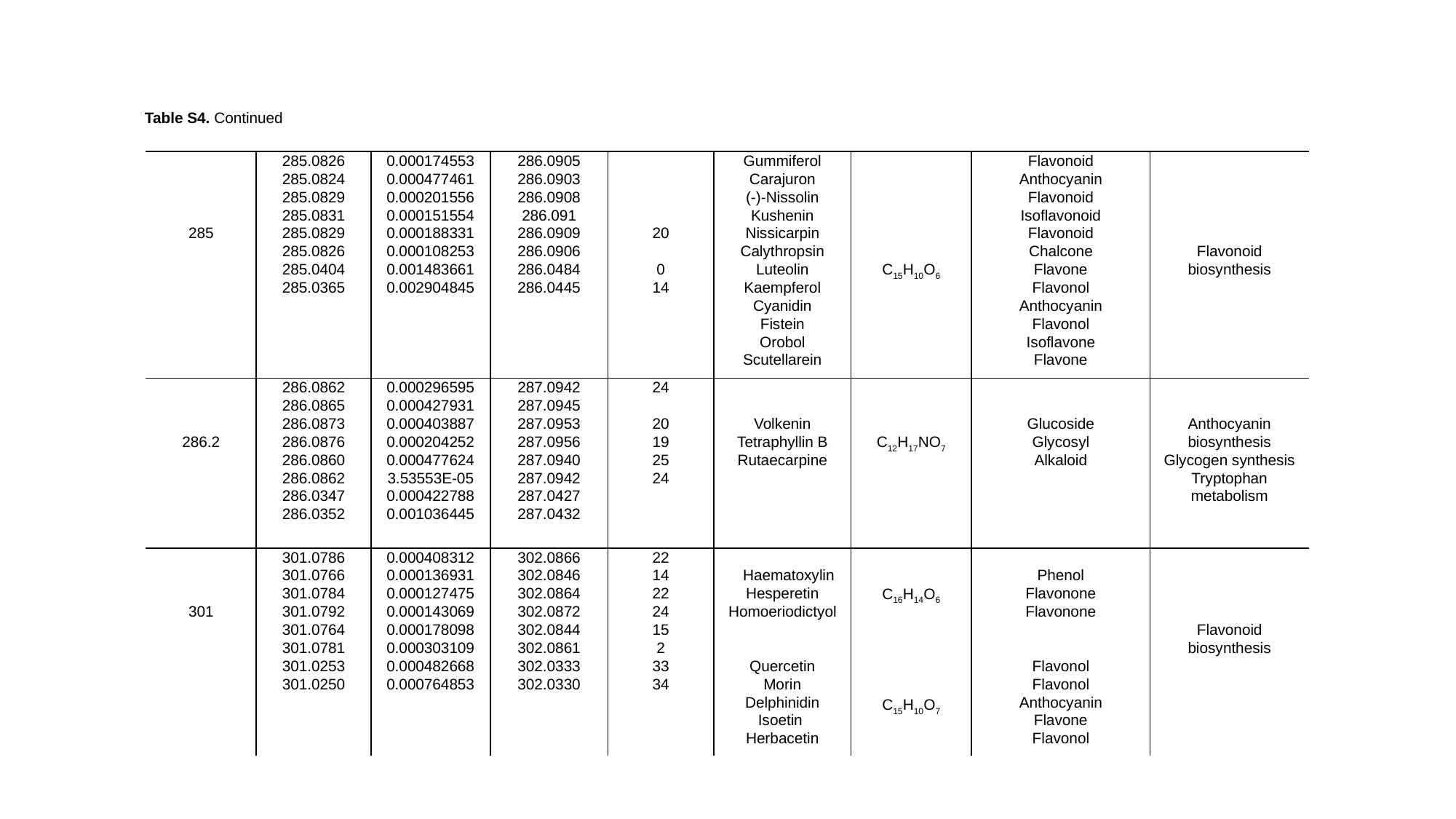

Table S4. Continued
| 285 | 285.0826 285.0824 285.0829 285.0831 285.0829 285.0826 285.0404 285.0365 | 0.000174553 0.000477461 0.000201556 0.000151554 0.000188331 0.000108253 0.001483661 0.002904845 | 286.0905 286.0903 286.0908 286.091 286.0909 286.0906 286.0484 286.0445 | 20   0 14 | Gummiferol Carajuron (-)-Nissolin Kushenin Nissicarpin Calythropsin Luteolin Kaempferol Cyanidin Fistein Orobol Scutellarein | C15H10O6 | Flavonoid Anthocyanin Flavonoid Isoflavonoid Flavonoid Chalcone Flavone Flavonol Anthocyanin Flavonol Isoflavone Flavone | Flavonoid biosynthesis |
| --- | --- | --- | --- | --- | --- | --- | --- | --- |
| 286.2 | 286.0862 286.0865 286.0873 286.0876 286.0860 286.0862 286.0347 286.0352 | 0.000296595 0.000427931 0.000403887 0.000204252 0.000477624 3.53553E-05 0.000422788 0.001036445 | 287.0942 287.0945 287.0953 287.0956 287.0940 287.0942 287.0427 287.0432 | 24   20 19 25 24 | Volkenin Tetraphyllin B Rutaecarpine | C12H17NO7 | Glucoside Glycosyl Alkaloid | Anthocyanin biosynthesis Glycogen synthesis Tryptophan metabolism |
| 301 | 301.0786 301.0766 301.0784 301.0792 301.0764 301.0781 301.0253 301.0250 | 0.000408312 0.000136931 0.000127475 0.000143069 0.000178098 0.000303109 0.000482668 0.000764853 | 302.0866 302.0846 302.0864 302.0872 302.0844 302.0861 302.0333 302.0330 | 22 14 22 24 15 2 33 34 | Haematoxylin Hesperetin Homoeriodictyol     Quercetin Morin Delphinidin Isoetin Herbacetin | C16H14O6           C15H10O7 | Phenol Flavonone Flavonone     Flavonol Flavonol Anthocyanin Flavone Flavonol | Flavonoid biosynthesis |

## Slide 6
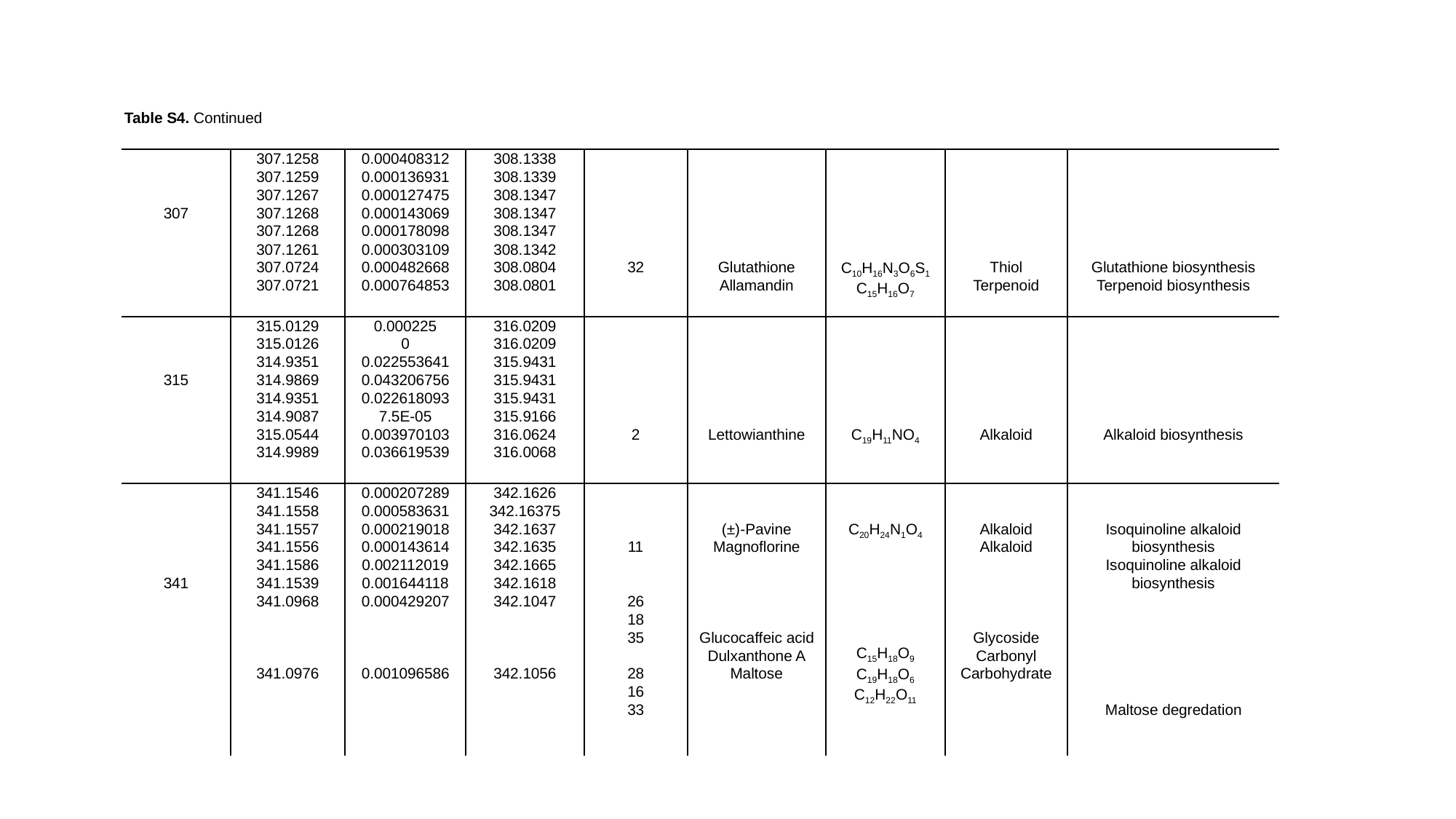

Table S4. Continued
| 307 | 307.1258 307.1259 307.1267 307.1268 307.1268 307.1261 307.0724 307.0721 | 0.000408312 0.000136931 0.000127475 0.000143069 0.000178098 0.000303109 0.000482668 0.000764853 | 308.1338 308.1339 308.1347 308.1347 308.1347 308.1342 308.0804 308.0801 | 32 | Glutathione Allamandin | C10H16N3O6S1 C15H16O7 | Thiol Terpenoid | Glutathione biosynthesis Terpenoid biosynthesis |
| --- | --- | --- | --- | --- | --- | --- | --- | --- |
| 315 | 315.0129 315.0126 314.9351 314.9869 314.9351 314.9087 315.0544 314.9989 | 0.000225 0 0.022553641 0.043206756 0.022618093 7.5E-05 0.003970103 0.036619539 | 316.0209 316.0209 315.9431 315.9431 315.9431 315.9166 316.0624 316.0068 | 2 | Lettowianthine | C19H11NO4 | Alkaloid | Alkaloid biosynthesis |
| 341 | 341.1546 341.1558 341.1557 341.1556 341.1586 341.1539 341.0968       341.0976 | 0.000207289 0.000583631 0.000219018 0.000143614 0.002112019 0.001644118 0.000429207       0.001096586 | 342.1626 342.16375 342.1637 342.1635 342.1665 342.1618 342.1047       342.1056 | 11     26 18 35   28 16 33 | (±)-Pavine Magnoflorine         Glucocaffeic acid Dulxanthone A Maltose | C20H24N1O4           C15H18O9 C19H18O6 C12H22O11 | Alkaloid Alkaloid         Glycoside Carbonyl Carbohydrate | Isoquinoline alkaloid biosynthesis Isoquinoline alkaloid biosynthesis             Maltose degredation |

## Slide 7
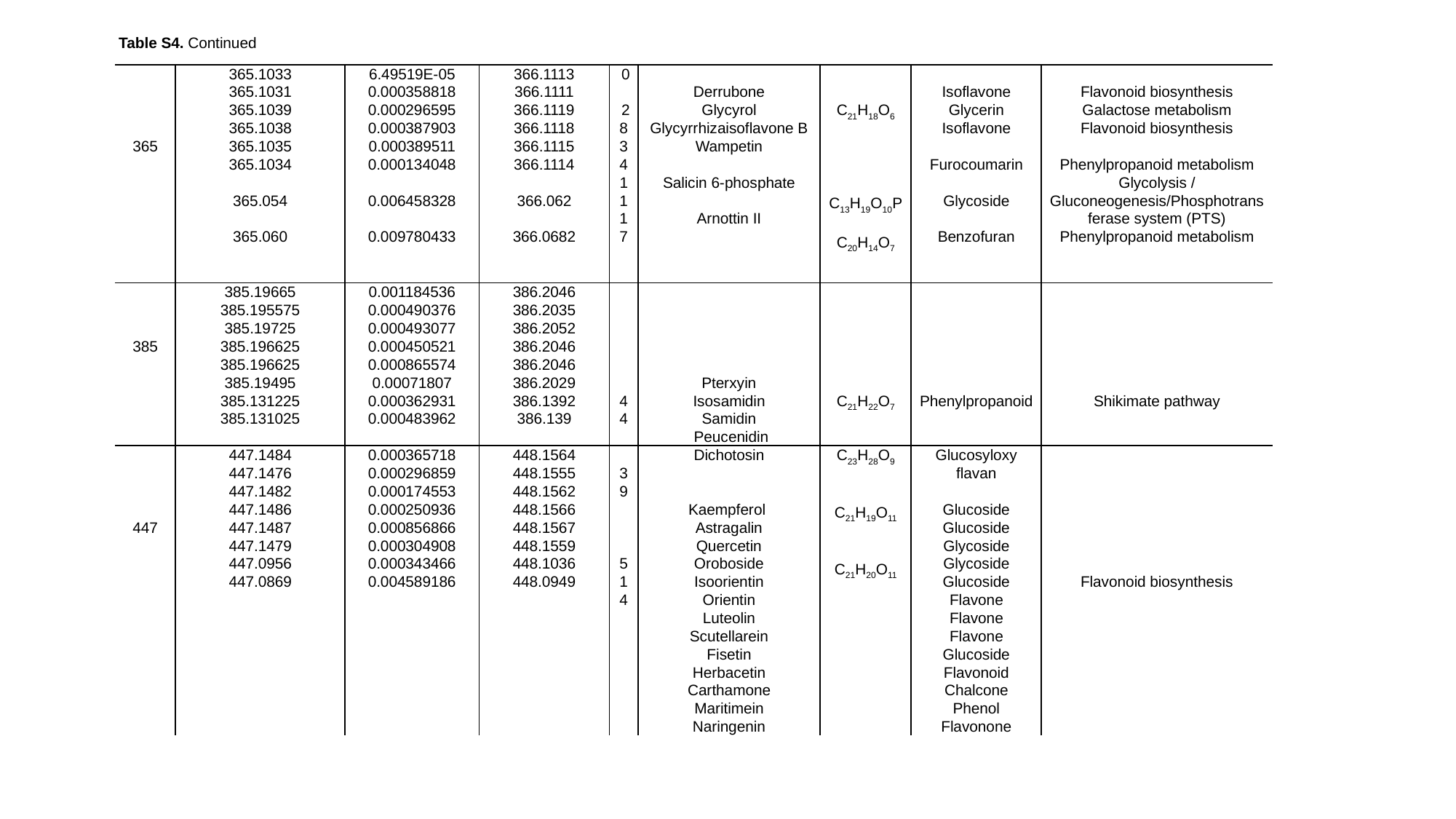

Table S4. Continued
| 365 | 365.1033 365.1031 365.1039 365.1038 365.1035 365.1034   365.054   365.060 | 6.49519E-05 0.000358818 0.000296595 0.000387903 0.000389511 0.000134048   0.006458328   0.009780433 | 366.1113 366.1111 366.1119 366.1118 366.1115 366.1114   366.062   366.0682 | 0    28 34 11 17 | Derrubone Glycyrol Glycyrrhizaisoflavone B Wampetin   Salicin 6-phosphate   Arnottin II | C21H18O6         C13H19O10P   C20H14O7 | Isoflavone Glycerin Isoflavone   Furocoumarin   Glycoside   Benzofuran | Flavonoid biosynthesis Galactose metabolism Flavonoid biosynthesis   Phenylpropanoid metabolism Glycolysis / Gluconeogenesis/Phosphotransferase system (PTS) Phenylpropanoid metabolism |
| --- | --- | --- | --- | --- | --- | --- | --- | --- |
| 385 | 385.19665 385.195575 385.19725 385.196625 385.196625 385.19495 385.131225 385.131025 | 0.001184536 0.000490376 0.000493077 0.000450521 0.000865574 0.00071807 0.000362931 0.000483962 | 386.2046 386.2035 386.2052 386.2046 386.2046 386.2029 386.1392 386.139 | 4 4 | Pterxyin Isosamidin Samidin Peucenidin | C21H22O7 | Phenylpropanoid | Shikimate pathway |
| 447 | 447.1484 447.1476 447.1482 447.1486 447.1487 447.1479 447.0956 447.0869 | 0.000365718 0.000296859 0.000174553 0.000250936 0.000856866 0.000304908 0.000343466 0.004589186 | 448.1564 448.1555 448.1562 448.1566 448.1567 448.1559 448.1036 448.0949 | 39       5 14 | Dichotosin     Kaempferol Astragalin Quercetin Oroboside Isoorientin Orientin Luteolin Scutellarein Fisetin Herbacetin Carthamone Maritimein Naringenin | C23H28O9     C21H19O11     C21H20O11 | Glucosyloxy flavan   Glucoside Glucoside Glycoside Glycoside Glucoside Flavone Flavone Flavone Glucoside Flavonoid Chalcone Phenol Flavonone | Flavonoid biosynthesis |

## Slide 8
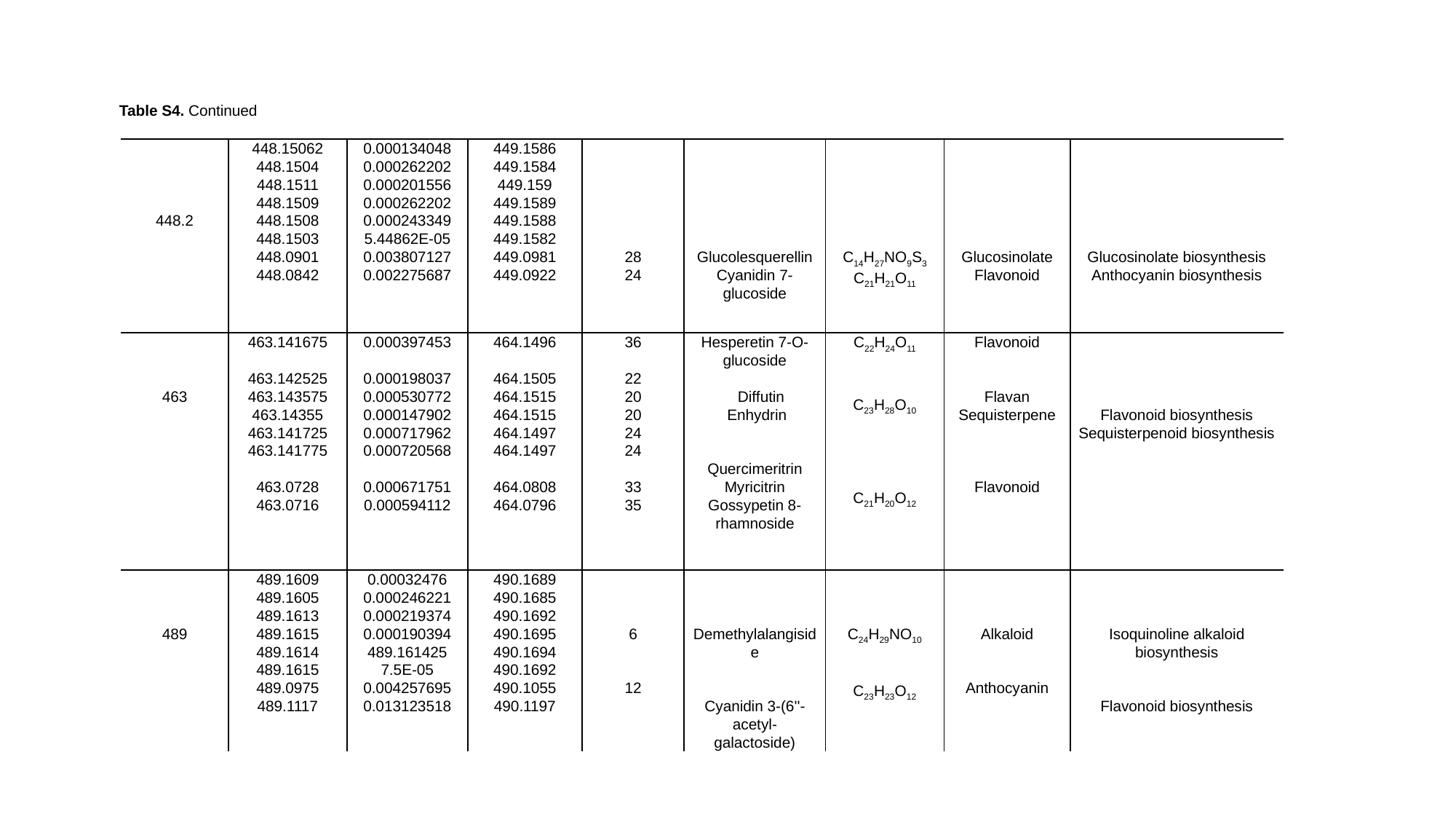

Table S4. Continued
| 448.2 | 448.15062 448.1504 448.1511 448.1509 448.1508 448.1503 448.0901 448.0842 | 0.000134048 0.000262202 0.000201556 0.000262202 0.000243349 5.44862E-05 0.003807127 0.002275687 | 449.1586 449.1584 449.159 449.1589 449.1588 449.1582 449.0981 449.0922 | 28 24 | Glucolesquerellin Cyanidin 7-glucoside | C14H27NO9S3 C21H21O11 | Glucosinolate Flavonoid | Glucosinolate biosynthesis Anthocyanin biosynthesis |
| --- | --- | --- | --- | --- | --- | --- | --- | --- |
| 463 | 463.141675   463.142525 463.143575 463.14355 463.141725 463.141775   463.0728 463.0716 | 0.000397453   0.000198037 0.000530772 0.000147902 0.000717962 0.000720568   0.000671751 0.000594112 | 464.1496   464.1505 464.1515 464.1515 464.1497 464.1497   464.0808 464.0796 | 36   22 20 20 24 24   33 35 | Hesperetin 7-O-glucoside      Diffutin Enhydrin     Quercimeritrin Myricitrin Gossypetin 8-rhamnoside | C22H24O11     C23H28O10         C21H20O12 | Flavonoid     Flavan Sequisterpene       Flavonoid | Flavonoid biosynthesis Sequisterpenoid biosynthesis |
| 489 | 489.1609 489.1605 489.1613 489.1615 489.1614 489.1615 489.0975 489.1117 | 0.00032476 0.000246221 0.000219374 0.000190394 489.161425 7.5E-05 0.004257695 0.013123518 | 490.1689 490.1685 490.1692 490.1695 490.1694 490.1692 490.1055 490.1197 | 6     12 | Demethylalangiside     Cyanidin 3-(6''-acetyl-galactoside) | C24H29NO10     C23H23O12 | Alkaloid     Anthocyanin | Isoquinoline alkaloid biosynthesis     Flavonoid biosynthesis |

## Slide 9
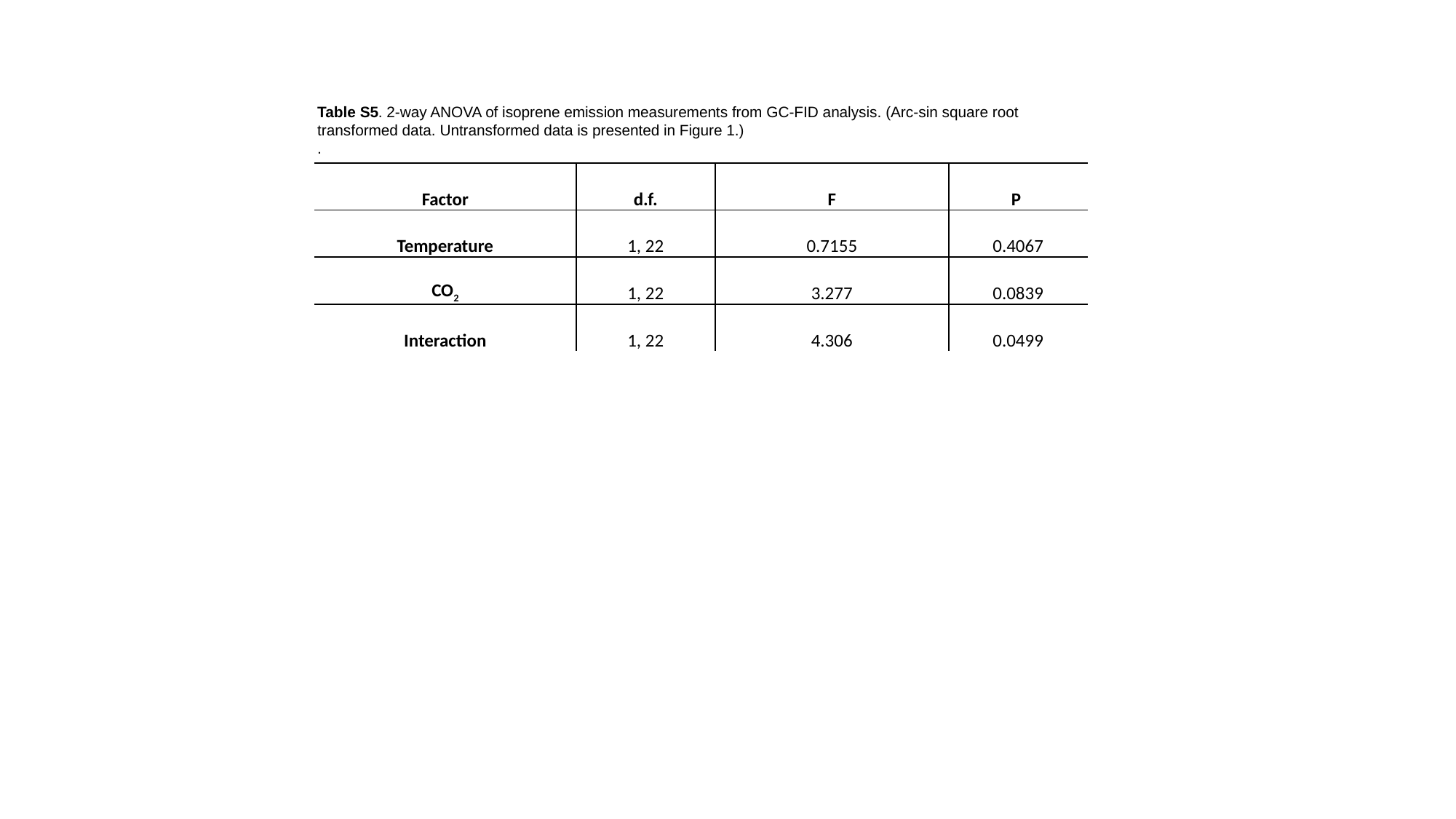

Table S5. 2-way ANOVA of isoprene emission measurements from GC-FID analysis. (Arc-sin square root transformed data. Untransformed data is presented in Figure 1.)
.
| Factor | d.f. | F | P |
| --- | --- | --- | --- |
| Temperature | 1, 22 | 0.7155 | 0.4067 |
| CO2 | 1, 22 | 3.277 | 0.0839 |
| Interaction | 1, 22 | 4.306 | 0.0499 |

## Slide 10
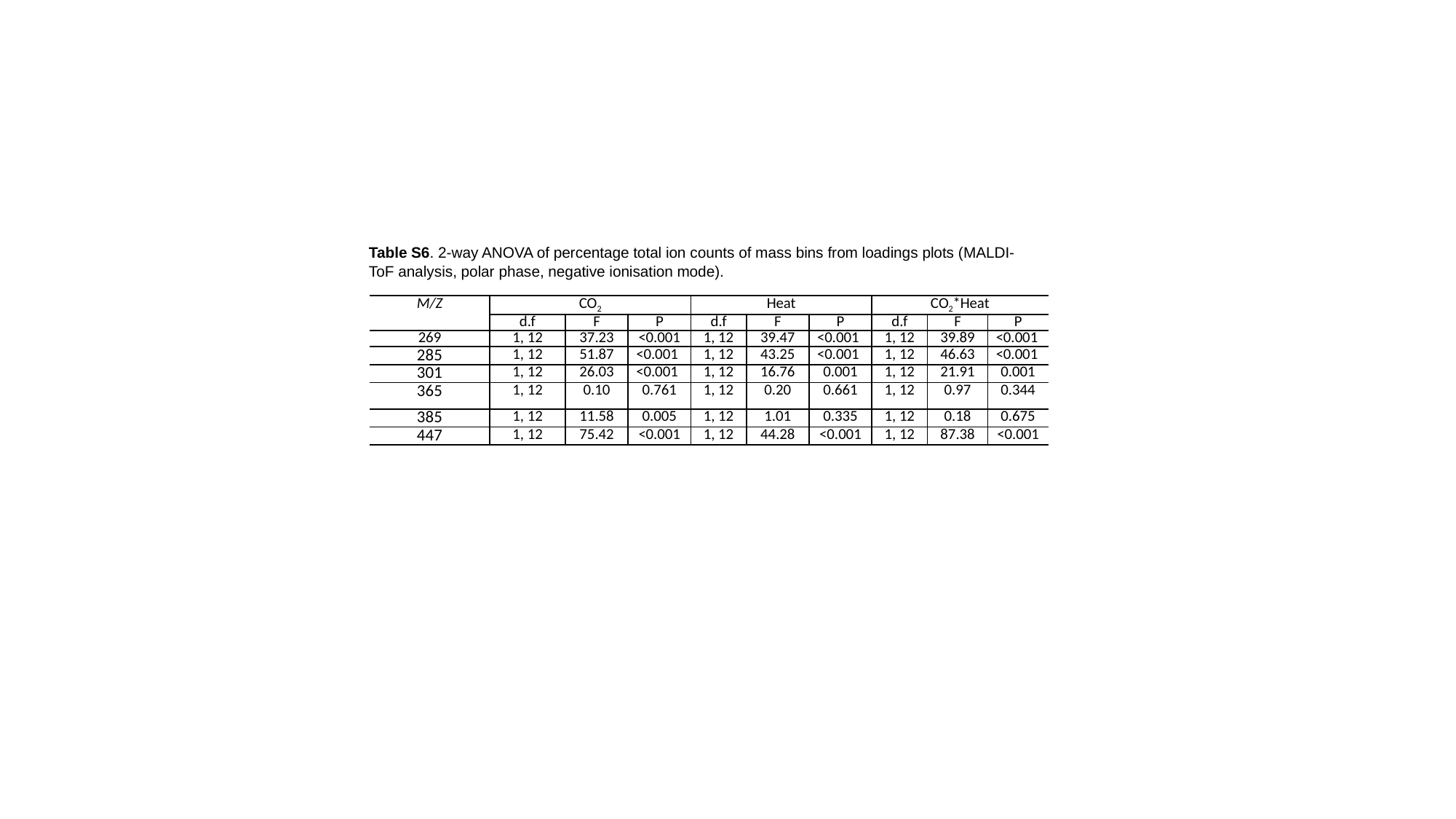

Table S6. 2-way ANOVA of percentage total ion counts of mass bins from loadings plots (MALDI-ToF analysis, polar phase, negative ionisation mode).
| M/Z | CO2 | | | Heat | | | CO2\*Heat | | |
| --- | --- | --- | --- | --- | --- | --- | --- | --- | --- |
| | d.f | F | P | d.f | F | P | d.f | F | P |
| 269 | 1, 12 | 37.23 | <0.001 | 1, 12 | 39.47 | <0.001 | 1, 12 | 39.89 | <0.001 |
| 285 | 1, 12 | 51.87 | <0.001 | 1, 12 | 43.25 | <0.001 | 1, 12 | 46.63 | <0.001 |
| 301 | 1, 12 | 26.03 | <0.001 | 1, 12 | 16.76 | 0.001 | 1, 12 | 21.91 | 0.001 |
| 365 | 1, 12 | 0.10 | 0.761 | 1, 12 | 0.20 | 0.661 | 1, 12 | 0.97 | 0.344 |
| 385 | 1, 12 | 11.58 | 0.005 | 1, 12 | 1.01 | 0.335 | 1, 12 | 0.18 | 0.675 |
| 447 | 1, 12 | 75.42 | <0.001 | 1, 12 | 44.28 | <0.001 | 1, 12 | 87.38 | <0.001 |

## Slide 11
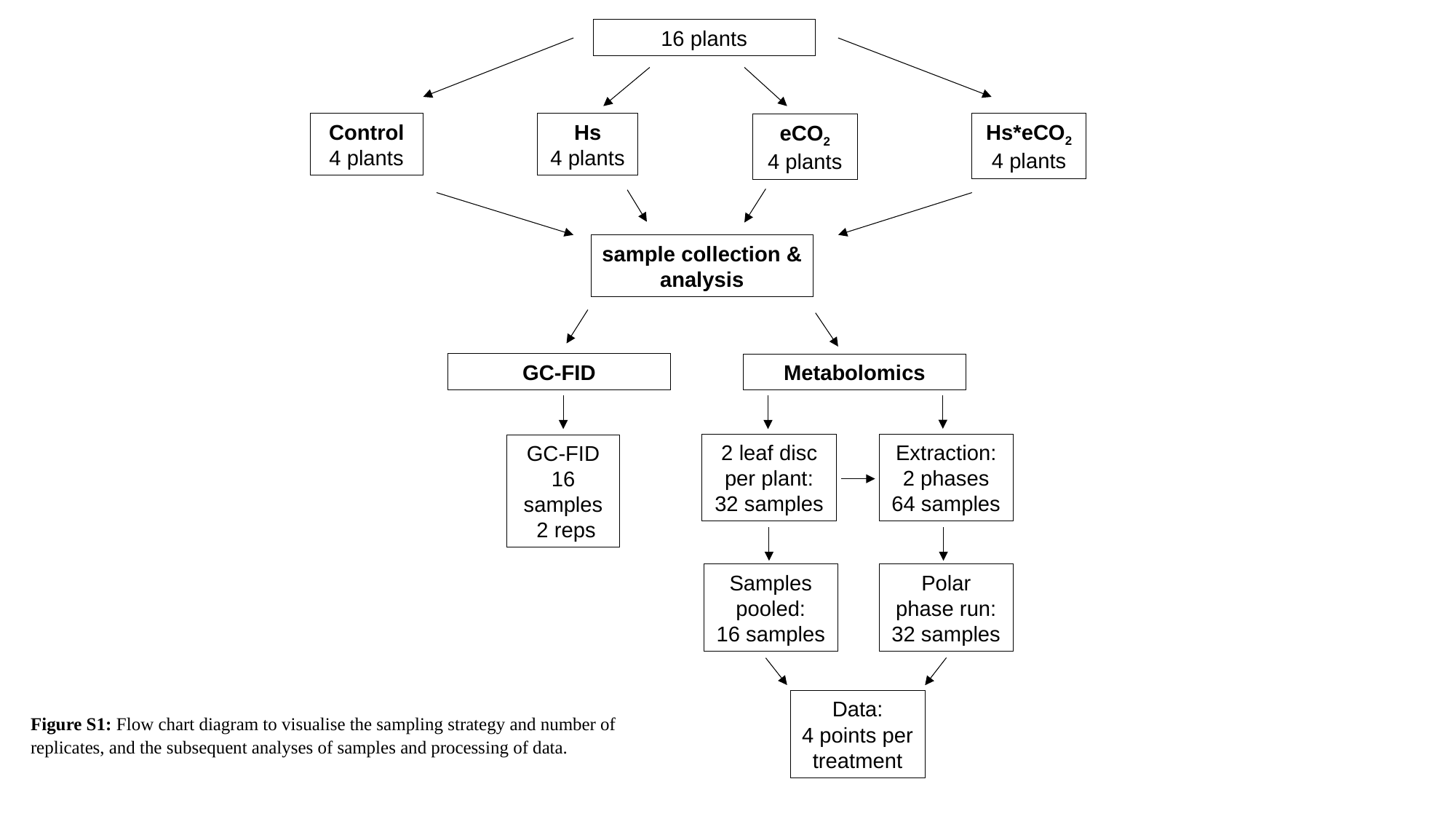

16 plants
Control
4 plants
Hs
4 plants
Hs*eCO2
4 plants
eCO2
4 plants
sample collection & analysis
GC-FID
Metabolomics
2 leaf disc per plant:
32 samples
Extraction: 2 phases
64 samples
GC-FID
16 samples
 2 reps
Samples pooled:
16 samples
Polar phase run:
32 samples
Data:
4 points per treatment
Figure S1: Flow chart diagram to visualise the sampling strategy and number of replicates, and the subsequent analyses of samples and processing of data.

## Slide 12
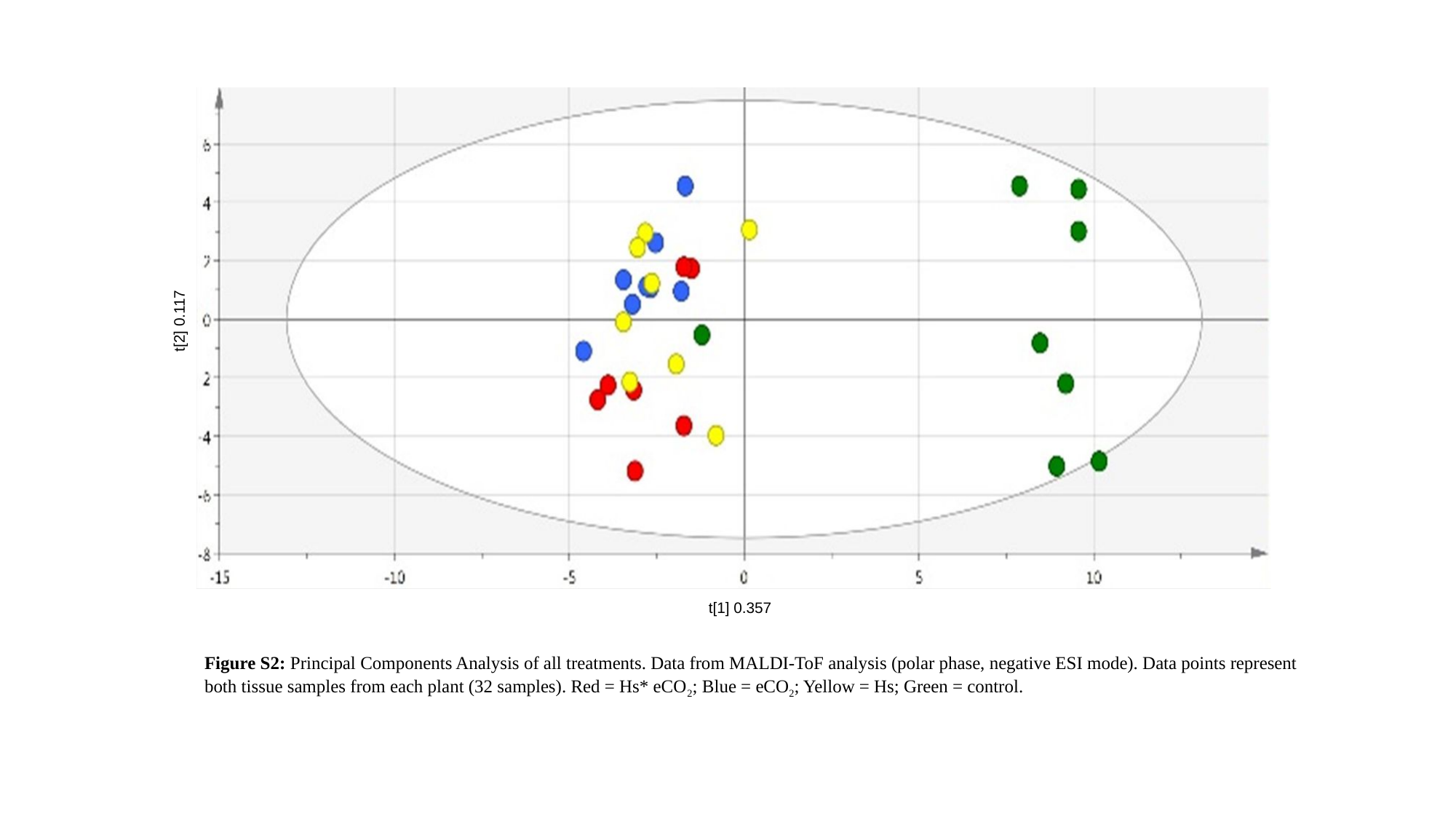

t[2] 0.117
t[1] 0.357
Figure S2: Principal Components Analysis of all treatments. Data from MALDI-ToF analysis (polar phase, negative ESI mode). Data points represent both tissue samples from each plant (32 samples). Red = Hs* eCO2; Blue = eCO2; Yellow = Hs; Green = control.

## Slide 13
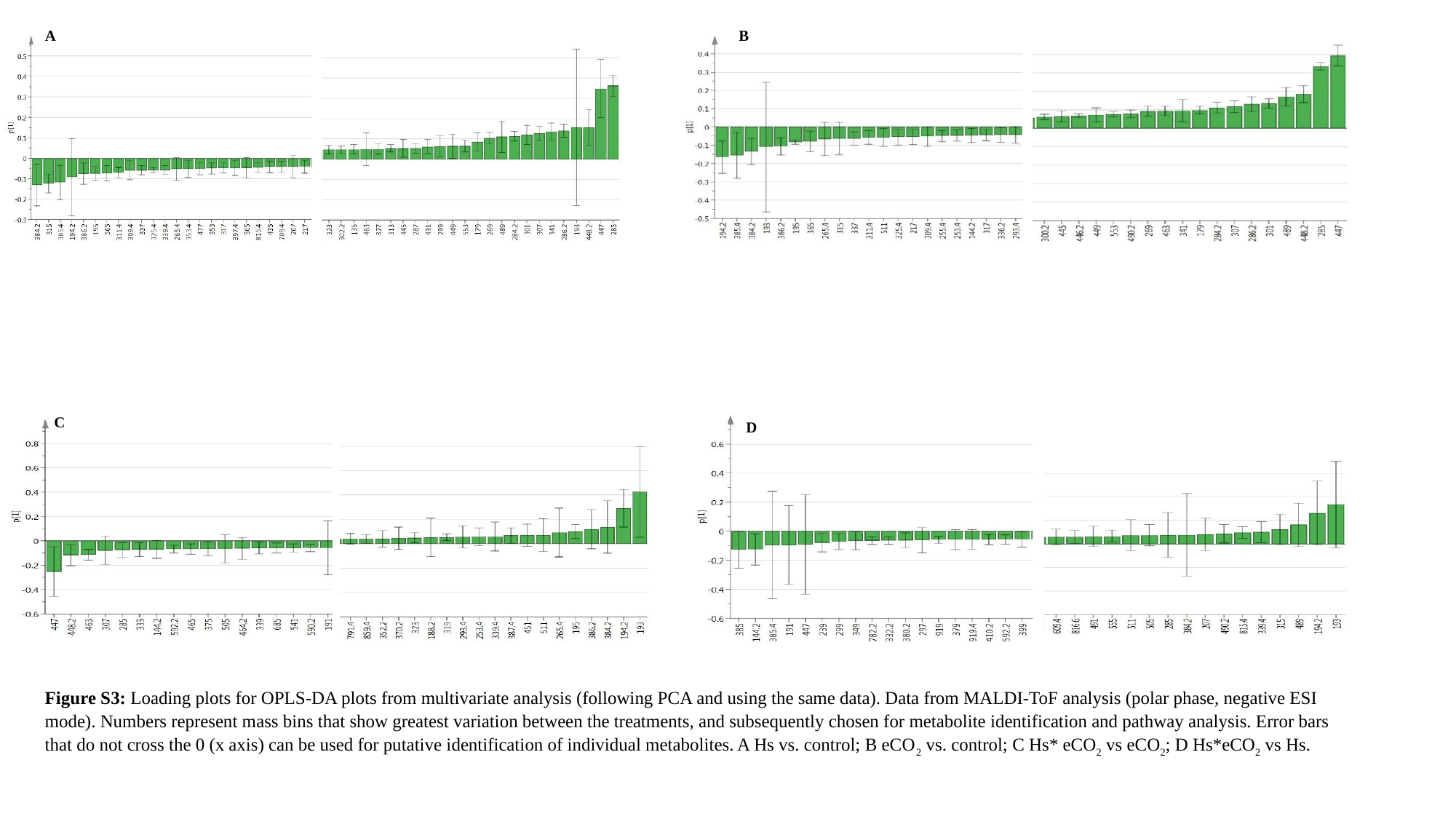

A
B
C
D
Figure S3: Loading plots for OPLS-DA plots from multivariate analysis (following PCA and using the same data). Data from MALDI-ToF analysis (polar phase, negative ESI mode). Numbers represent mass bins that show greatest variation between the treatments, and subsequently chosen for metabolite identification and pathway analysis. Error bars that do not cross the 0 (x axis) can be used for putative identification of individual metabolites. A Hs vs. control; B eCO2 vs. control; C Hs* eCO2 vs eCO2; D Hs*eCO2 vs Hs.

## Slide 14
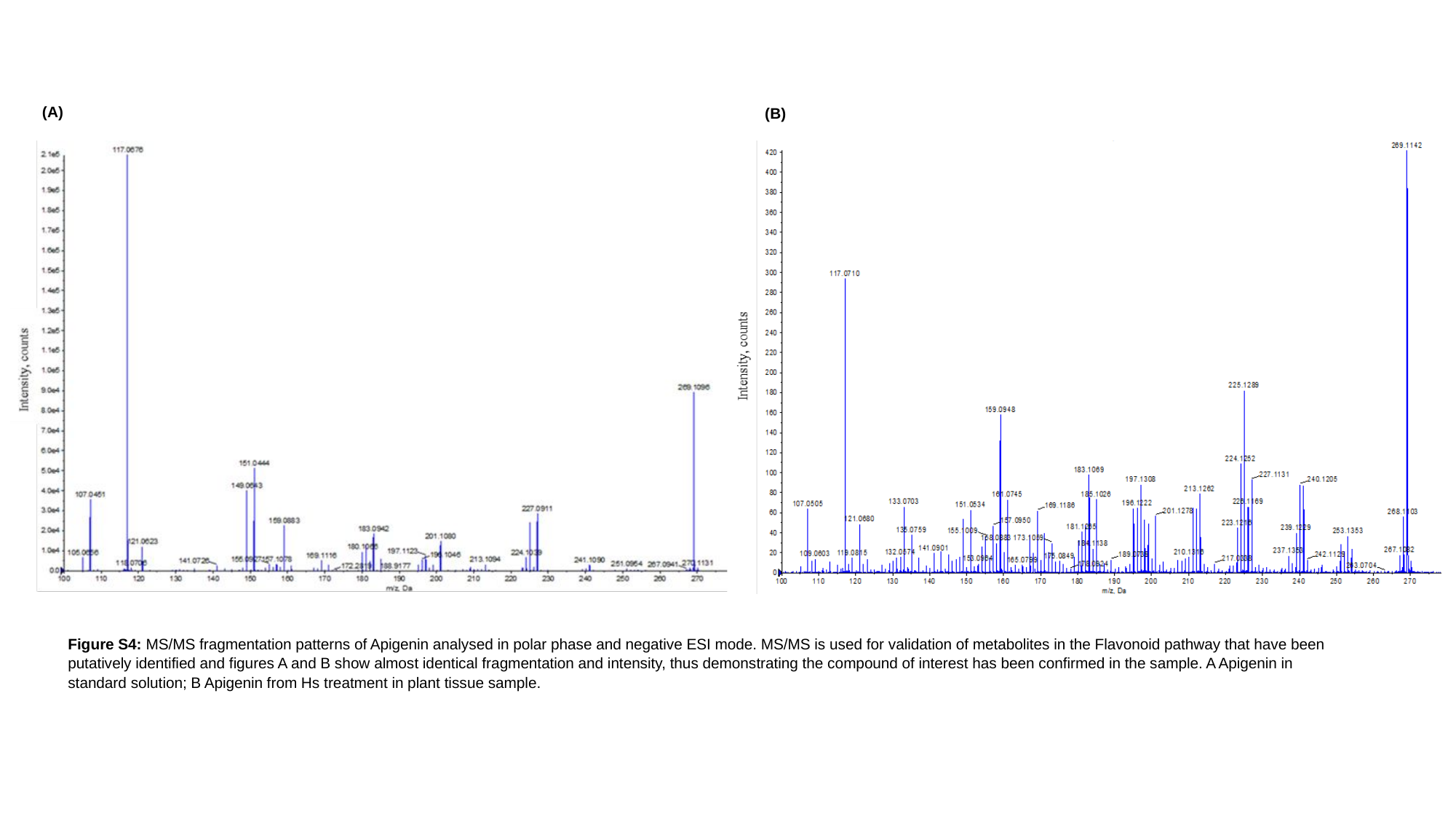

(A)
(B)
Figure S4: MS/MS fragmentation patterns of Apigenin analysed in polar phase and negative ESI mode. MS/MS is used for validation of metabolites in the Flavonoid pathway that have been putatively identified and figures A and B show almost identical fragmentation and intensity, thus demonstrating the compound of interest has been confirmed in the sample. A Apigenin in standard solution; B Apigenin from Hs treatment in plant tissue sample.

## Slide 15
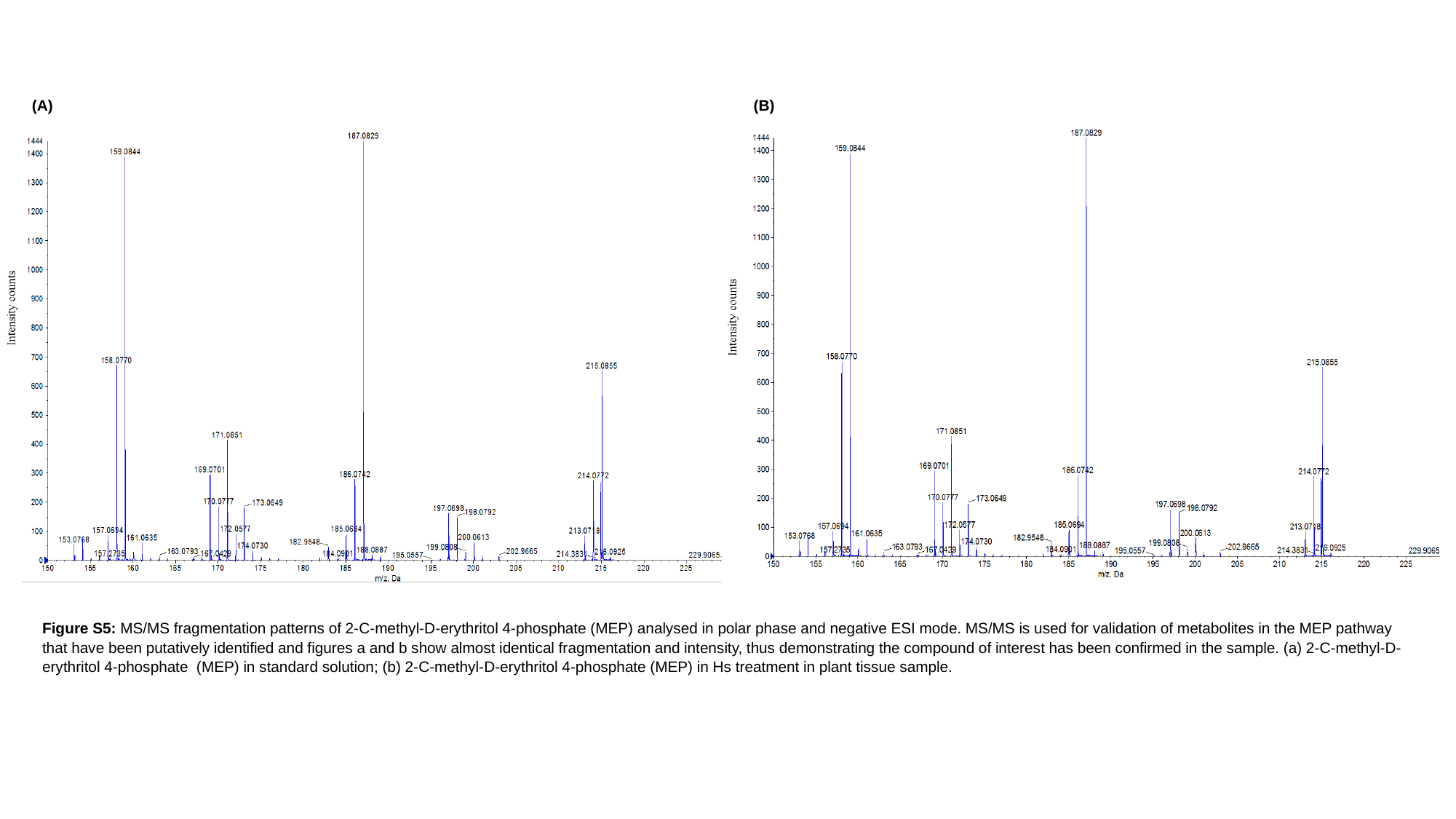

(A)
(B)
Figure S5: MS/MS fragmentation patterns of 2-C-methyl-D-erythritol 4-phosphate (MEP) analysed in polar phase and negative ESI mode. MS/MS is used for validation of metabolites in the MEP pathway that have been putatively identified and figures a and b show almost identical fragmentation and intensity, thus demonstrating the compound of interest has been confirmed in the sample. (a) 2-C-methyl-D-erythritol 4-phosphate (MEP) in standard solution; (b) 2-C-methyl-D-erythritol 4-phosphate (MEP) in Hs treatment in plant tissue sample.
